# Warming Climate Is Reducing the Diversity of Dominant Microbes in the Largest High Arctic Lake

**DOI:** 10.3389/fmicb.2020.561194

**Published:** 2020-10-07

**Authors:** Graham A. Colby, Matti O. Ruuskanen, Kyra A. St.Pierre, Vincent L. St.Louis, Alexandre J. Poulain, Stéphane Aris-Brosou

**Affiliations:** ^1^Department of Biology, University of Ottawa, Ottawa, ON, Canada; ^2^Department of Biological Sciences, University of Alberta, Edmonton, AB, Canada; ^3^Department of Mathematics and Statistics, University of Ottawa, Ottawa, ON, Canada

**Keywords:** high arctic, microbial ecology, metagenome assembled genomes (MAGs), high-throughput sequencing, climate change

## Abstract

Temperatures in the Arctic are expected to increase dramatically over the next century, and transform high latitude watersheds. However, little is known about how microbial communities and their underlying metabolic processes will be affected by these environmental changes in freshwater sedimentary systems. To address this knowledge gap, we analyzed sediments from Lake Hazen, NU Canada. Here, we exploit the spatial heterogeneity created by varying runoff regimes across the watershed of this uniquely large high-latitude lake to test how a transition from low to high runoff, used as one proxy for climate change, affects the community structure and functional potential of dominant microbes. Based on metagenomic analyses of lake sediments along these spatial gradients, we show that increasing runoff leads to a decrease in taxonomic and functional diversity of sediment microbes. Our findings are likely to apply to other, smaller, glacierized watersheds typical of polar or high latitude ecosystems; we can predict that such changes will have far reaching consequences on these ecosystems by affecting nutrient biogeochemical cycling, the direction and magnitude of which are yet to be determined.

## Introduction

Climate change is amplified1 in polar regions, where near-surface temperatures have increased almost twice as fast as elsewhere on Earth over the last decade (Overpeck et al., 1997; Serreze and Francis, 2006; Screen and Simmonds, 2010). Climate models predict that annual surface temperatures will increase in the Arctic by as much as 8°C by 2100 relative to the 1981–2010 average (Mingle, 2020). These changes are already having dramatic consequences on physical (Bliss et al., 2014; O'Reilly et al., 2015; Laudon et al., 2017), biogeochemical (Frey and McClelland, 2009; Lehnherr et al., 2018), and ecological (Smol et al., 2005; Wrona et al., 2016) processes across Arctic ecosystems. During the winter months (January–March) of 2016 and 2018 surface temperature in the central Arctic were already 6°C warmer (Mingle, 2020). While we are starting to understand the effect of thawing permafrost on microbial communities and shallow aquatic ecosystems (McCalley et al., 2014; Crevecoeur et al., 2015; Hultman et al., 2015; Mackelprang et al., 2016), our knowledge of how microbial communities in large aquatic ecosystems at high latitudes respond to environmental changes is comparatively lacking in part due to the large spatial variability to be expected in such systems. Furthermore, lakes are broadly considered sentinels of climate change, as they integrate physical, chemical, and biological changes happening through their watersheds (Williamson et al., 2009); however, their microbial community structure and function are relatively understudied, in particular in the Arctic.

To date, much of the research performed on microbial communities in Arctic lakes has been limited to studies that were mostly based on partial 16S rRNA gene sequencing (Stoeva et al., 2014; Mohit et al., 2017; Thaler et al., 2017; Ruuskanen et al., 2018; Cavaco et al., 2019). While these studies are useful to understand the structure of these microbial communities, they provide limited functional insights and can be biased as they often rely on sequence databases where environmental microbes, specifically from the Arctic, may be underrepresented (Ruuskanen et al., 2018, 2019). More critically, being circumscribed both in space and in time, previous studies only offer snapshots of microbial communities and hence, have a limited power to predict how microbial communities might respond to climate change.

To predict the effect of climate change on microbial functional diversity in Arctic lake sediments, we focused on Lake Hazen, the world's largest High Arctic lake (82° N, 71°W) by volume which is estimated at 5.14 × 10^10^ m^3^ (Köck et al., 2012). Lake Hazen is ultra-oligotrophic, and its physical, chemical, and biological limnology has been previously described in depth in St.Pierre et al. (2019b). In this work, we exploited two important properties of Lake Hazen. First, its watershed is already experiencing the effects of climate change, as increasing regional temperatures are leading to more glacial melt, permafrost thaw, and increased runoff from the watershed into the lake in warmer years relative to cooler ones (Lehnherr et al., 2018). Second, its tributaries are highly heterogeneous, fed by 11 glaciers ranging from 6 to 1,041 km^2^ in surface area, and annual runoff volume approximately scaling with their size (from <0.001 to 0.080 km^3^ in 2016; St.Pierre et al., 2019b). Glacial meltwaters feed the lake from late June through to the end of August.

**Figure 1:**
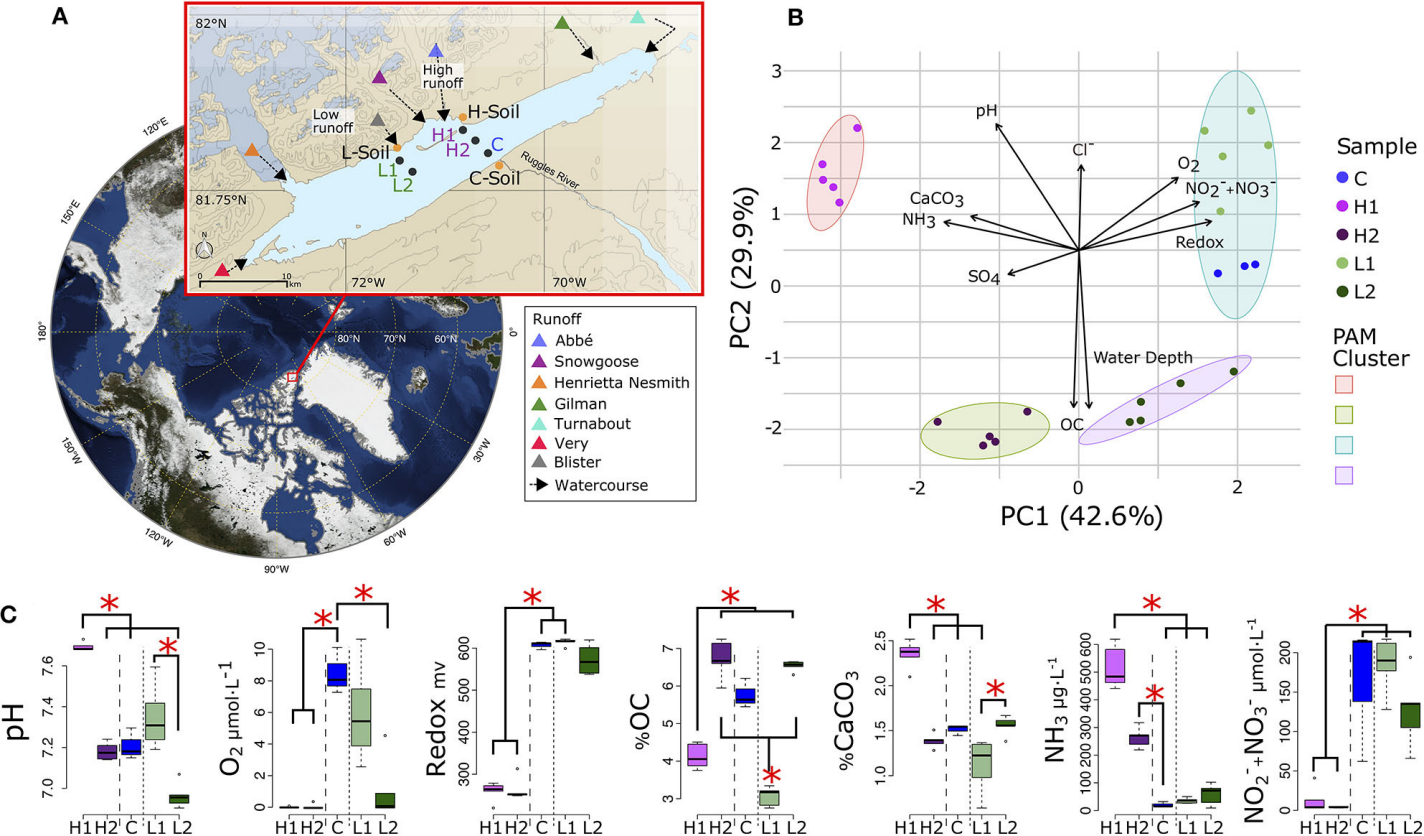
Lake Hazen sampling design and chemical composition. **(A)** Location of Lake Hazen (red box). Inset map: soil (orange dots) and sediment (black dots) sample sites are separated into hydrological regimes of high (purple), low (green), and negligible/control (blue) runoff. **(B)** Principal component analysis (PCA) showing the differences in physical and chemical composition of the sediment sites. Vectors display pH, dissolved dioxygen (O_2_), redox potential, nitrates and nitrites concentration (${\text{NO}}_{2}^{-}$ + ${\text{NO}}_{3}^{-}$), water depth, percent organic carbon (OC), percent calcium carbonate (CaCO_3_), sulfate (${\text{SO}}_{4}^{2-}$) concentration (SO_4_), and ammonia concentration (NH_3_). Individual points represent the mean values using 1 cm intervals measured in the top 5 cm. Partitioning around medoids was used to identify clusters. **(C)** Distribution of chemical features for sediment sites. Branches and asterisks indicate significant differences between sites *P* < 0.025 (Dunn Test). If branch tips form a dichotomy or trichotomy, the interactions within that group is not significant. Long dashes separate high runoff sites and dotted line separates low runoff sites. There was insufficient data to include soil sites in **(B,C)**.

It is this spatial heterogeneity in runoff that we used to evaluate the possible consequences of climate change on microbial structure and functional diversity in High Arctic sediment, acknowledging that the consequences of increasing temperature are likely more plural and complex. Although this approach simplifies the effects of climate change, glacial runoff provides the main source of sediment and nutrients to this extremely oligotrophic environment (Lehnherr et al., 2018). Therefore, increasing temperature and subsequent runoff are assumed to be the primary drivers of alterations to this microbial ecosystem. To this effect, we sampled lake sediments along two transects representing low (L transect: samples L1 [shallow] and L2 [deep]) and high (H: samples H1 [shallow] and H2 [deep]) seasonal runoff volume, as well as at a single site that received negligible runoff (C site; Figure 1A). In order to assess the potential level of connectivity between lake sediments and upstream soil (Crump et al., 2012; Comte et al., 2018; Hermans et al., 2020), we also collected soil samples (S sites) from three sites in the dried streambeds of the tributaries, on the northern shore between the two transects to assess soil influence on microbial communities present in the sediments. We then leveraged untargeted metagenomics analyses to draw an inventory of dominant microbes, assumed to be the most critical to nutrient cycling and the most relevant to the dynamics of microbial communities. These reconstructed Metagenome Assembled Genomes (MAGs) (Bowers et al., 2017) allowed us to assess the quantitative impact of a change of runoff regime, from low to high, on both the structure of sediment microbial communities and their functional potential. We show that an increase in runoff volume and resultant sedimentation rates, as predicted under climate change scenarios for the region, could lead to a reduced diversity of the dominant microbial community and of their functional potential.

## Results

###  Characterization of the Physical and Geochemical Environments

We first characterized how geochemical properties of the sediments varied along and between the two transects (soil chemistry could not be obtained due to the limited number of cores that could be collected and shipped back, and to in-lab restrictions on chemical measurements) by re-analyzing data from St.Pierre et al. (2019b). Sediment samples from these five sites clustered into four distinct geochemical groups (Figure 1B) that reflect spatial variability in glacial runoff, the primary hydrological input to the lake. Indeed, PC1 explained 43% of the total variance, and differentiated the L and high H runoff transects, while PC2 (29.9%) separated each transect according to their depth.

Along PC1, higher concentrations of ammonia (NH_3_) and sulfate (${\text{SO}}_{4-}^{2-}$) in the porewaters, and a greater percentage of calcium carbonate in the sediments, were present in the H transect. However, higher concentrations of dioxygen (O_2_), nitrates/nitrites (${\text{NO}}_{3}^{-}$/${\text{NO}}_{2}^{-}$), and greater redox potential were present in the L transect and the control (C) sites. Along PC2, sediment organic carbon (OC), and porewater pH and Cl^−^, were more determinant when discriminating between the shallow (L1 and H1) and deep (L2 and H2) sites of both transects. Rather than grouping spatially with the H transect, the C sites were most chemically similar to L1 (Figure 1C, Supplementary Figure 1). The shallow sites were not significantly different from each other in pH measurements or OC concentrations, but were both significantly different from the deeper sites suggesting that although most chemical features were similar within each transect, some features might still be influenced by their spatial proximity to the shoreline or depth of the overlying water column (Figure 1C).

###  Contrasting Low vs. High Runoff Transects Revealed a Decrease in Biodiversity

**Figure 2:**
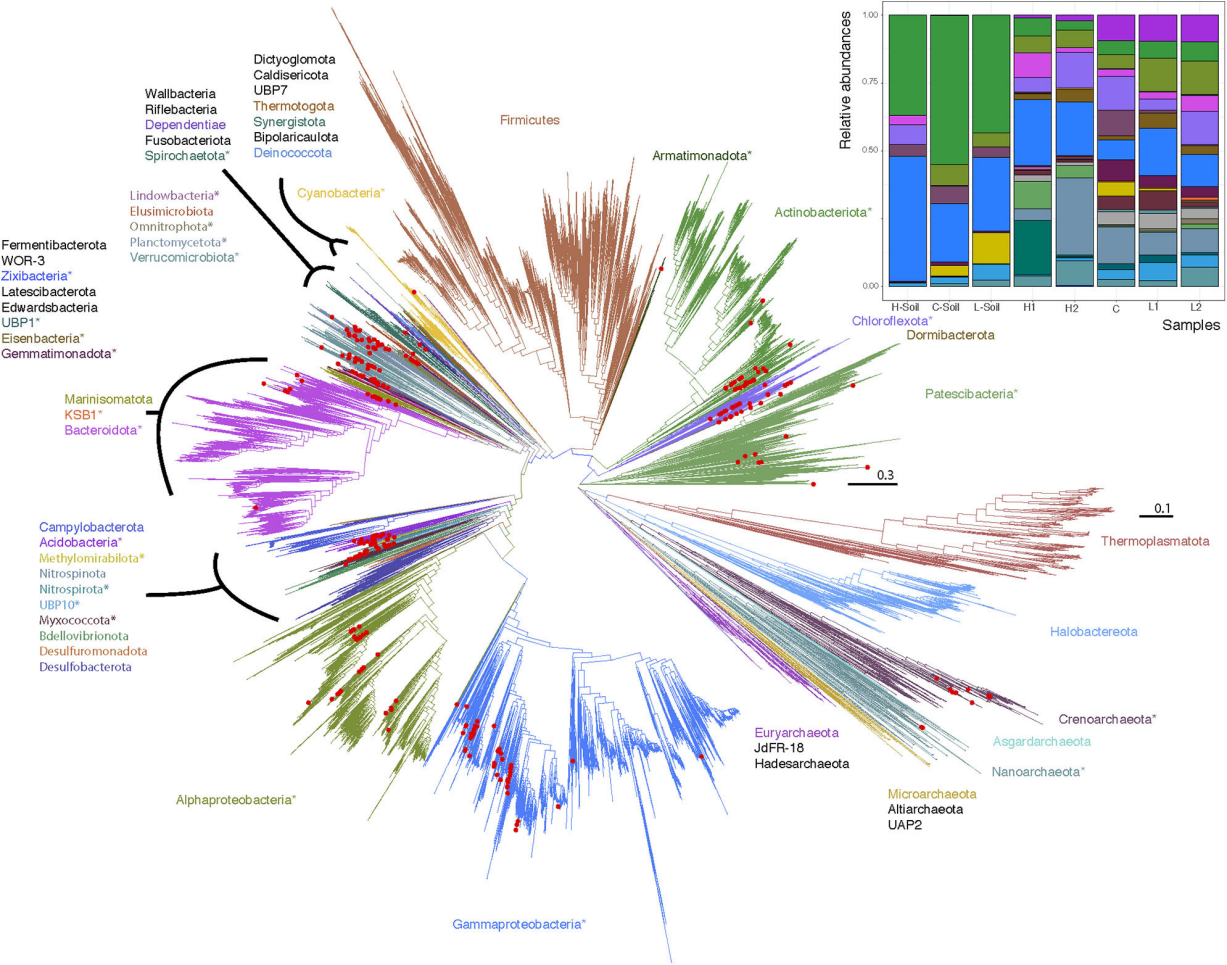
Maximum likelihood phylogenetic trees of Lake Hazen genomes based on 120 concatenated bacteria and 122 concatenated archaea protein-coding genes. Red Dots: Lake Hazen genomes. Asterisks (*) indicate phyla that contain Lake Hazen genomes. Bacteria tree is rooted with Patescibacteria and Archaea tree is rooted with Euryarchaeota. See GitHub account for full taxonomy tree files and for original tree files (Supplemental Data File 2 and 3) including the GTDB (v86) reference sequences (Parks et al., 2018). Inset shows MAG abundance across sites, in the 300 high quality genomes for each sample normalized to 100%.

With such a clear geochemical separation of the transects along PC1 (43% of explained variance) and significant spatial contrasts (Figure 1C), we had the right context to evaluate the influence of runoff gradients on sediment microbial diversity. We assembled a total of 300 (290 bacterial and 10 archaeal) MAGs that were >50% complete and with <10% contamination (Minimum information about a metagenome-assembled genome [MIMAG] guidelines; Bowers et al., 2017, Supplementary Tables 1, 2). By constructing phylogenetic trees for Bacteria and Archaea, we noted that while most major phyla were represented in the MAGs, no Firmicutes and only a small number of Archaea were identified (Figure 2). In contrast, Gammaproteobacteria (*n* = 50), Actinobacteria (*n* = 31), Alphaprobacteria (*n* = 24), Chloroflexoata (*n* = 30), Planctomycetota (*n* = 24), and Acidobacteriota (*n* = 19) were the most commonly recovered taxa across the entire watershed. Uncultured phyla comprised ~11% of reconstructed MAGs, including representatives from multiple taxa: Eisenbacteria (*n* = 12), Patescibacteria (*n* = 9), Omnitrophica (*n* = 5), KSB1 (*n* = 1), Armatimonadota (*n* = 1), Lindowbacteria (*n* = 1), USBP1 (*n* = 1), UBP10 (*n* = 1), and Zixibacteria (*n* = 1). We note that missing sequence data in the gene alignments had a minimal impact on the trees (Supplementary Text, Supplementary Figures 2, 3).

**Figure 3:**
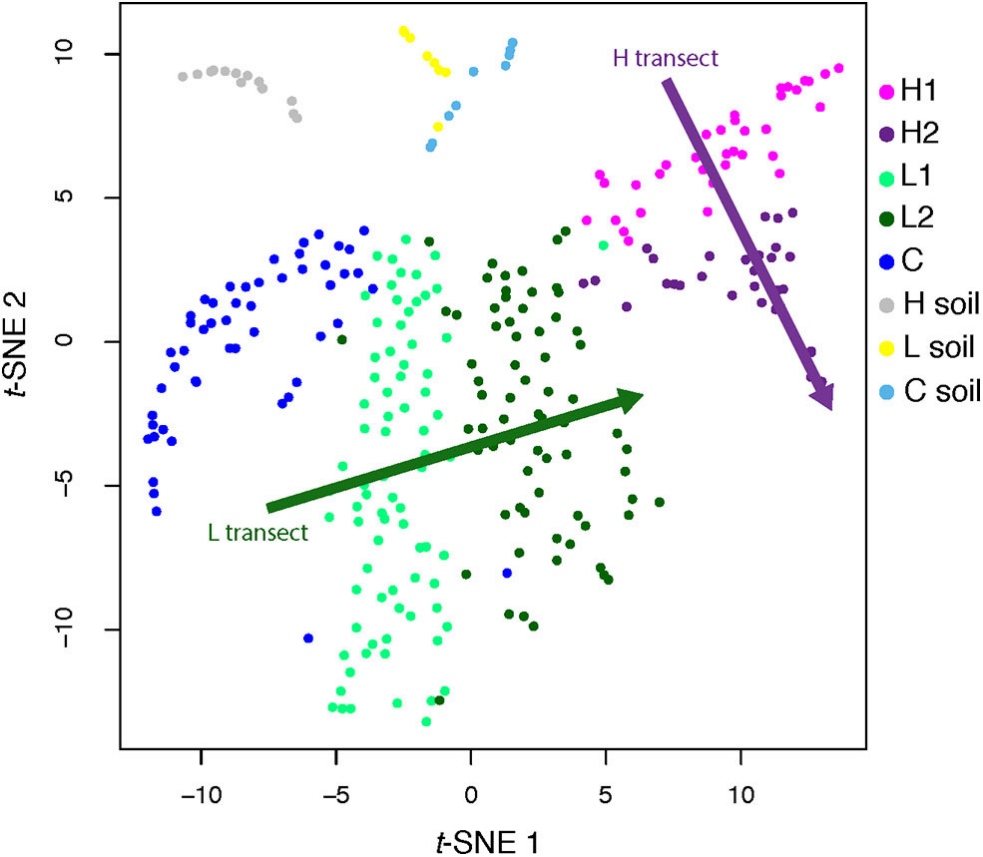
*t*-SNE analysis of genome abundance for each sediment sample. Each of the 300 shown genomes was assigned to the sample where it has the greatest abundance. Shaded arrows display the approximate direction of water flow, from upstream to downstream, for the high (green) and low (purple) transects. Note that these arrows do not indicate any kind of convergence.

However, these MAGs were not evenly distributed across all sites (Figure 2, inset; Supplementary Figure 4). To quantify this uneven distribution, we determined the site where each genome was most abundant. Based solely on this information, we performed an unsupervised clustering (*t*-SNE), and found that the directions defined by sediment-laden water flowing from the shallow to the deep site within each transect in the projection space were almost orthogonal between transects (see arrows in Figure 3, showing the relative positions of the hyperplanes defined by water flow)—which suggests that, as in a Principal Component Analysis, these directions are independent (although a *t*-SNE cannot strictly speaking be interpreted in this way), and hence that transitioning from the L to H transect leads to a dramatic shift in microbial communities. This shift can also be characterized from a diversity point of view, where both PCoA (Supplementary Figure 5) and a DPCoA (Supplementary Figure 6) results suggest differences between the L and H transects—at least from a qualitative standpoint.

**Figure 4:**
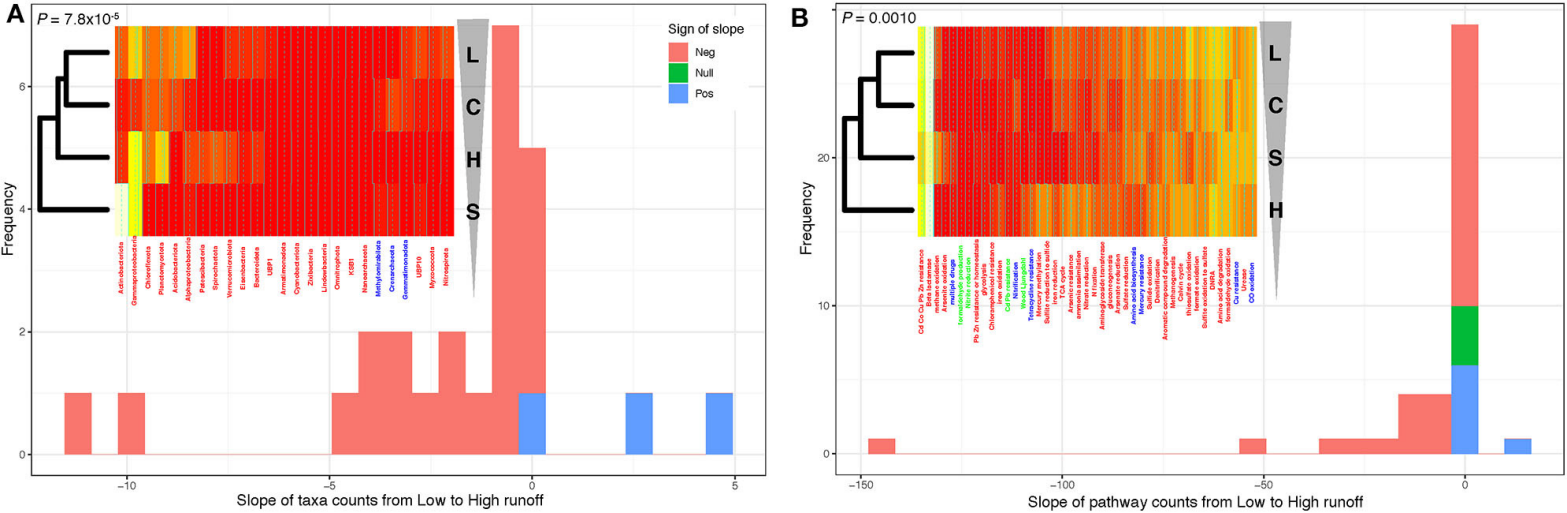
Transition from low to high runoff leads to a decrease in diversity. **(A)** Distribution of the slopes of taxonomic counts as a function of sites. **(B)** Distribution of the slopes of pathway counts as a function of sites. In both cases, counts were aggregated by location types (L [Low], C [Control], S [Soil], and H [High] sites), and linear models (ANOVA) were fitted to estimate the slope of each regression. Insets: heatmap representations of count tables (from white [low counts] to red [high]); leftmost dendrograms (hierarchical clustering) show how the location types cluster, transitioning from L to H runoffs (vertical triangle pointing down). Note that these dendrograms are “unrooted,” which means that in both panels, L and C one the one hand and H and S on the other hand cluster together. *P*-values: one-sided binomial test for enrichment in negative slopes across the 25 phyla. See Supplementary Figure 7 for additional details.

To quantify the direction and the significance of these structural and diversity shifts, we focused on the phylum level (to extract large-scale patterns), and calculated the relative proportions of each of the reconstructed 300 MAGs at each site, and tallied these numbers by phylum, over the 25 phyla represented in our data. We did this along each transect—essentially pooling sites H1/H2 together to represent the H transect, and doing the same for sites L1/L2 (the L transect), while keeping proportions for the S and C sites separate. Note that sites S and C were included at this stage to gauge the potential level of connectivity between lake sediments and both upstream and downstream soils. Hierarchical clustering on this table of MAGs proportions by phyla vs. sites showed a divergence from the L to H transects [following the (((L,C),H),S) clustering pattern; Figure 4A, inset—see also Supplementary Figures 7, 8], with the C site (negligible runoff) grouping with the L sites, the S sites (soils) with the H sites, and confirming the clear contrast between the two transects in terms of taxa proportions (see Figure 3). To test if these taxa proportions tended to increase or decrease when transitioning from L to H along the (((L,C),H),S) clustering pattern, we fitted linear models (ANOVA) regressing the proportions of each of the 25 phyla against sites, ordered as per their hierarchical clustering (L→C→H→S). Essentially, we regressed a single data point for each of the four classes (L, C, H, and S), so that *P*-values could not be obtained, but slope could be estimated (Figure 4A). Strikingly, most of these slopes were negative (22 out of 25; binomial test: *P* = 7.8 × 10^−8^; see also Supplementary Figure 8A), demonstrating a significant decrease in diversity at the phylum level as one goes from low to high runoff regimes. Because the inclusion of the C and S sites in these analyses could add noise when specifically testing for a change in the proportion of phyla during a L to H transition, we reran these analyses without the C and S sites. In spite of some phyla changing slope sign, suggesting a certain instability in our results due to small sample size, we found that most of the estimated slopes were negative (19 out of 25; binomial test: *P* = 0.0073; see also Supplementary Figure 8B), still providing evidence of a significant decrease in diversity at the phylum level as one goes from low to high runoff regimes.

An NMDS ordination allowed us to detect the geochemical features associated with this shift in microbial communities (Supplementary Figure 9). In the sediments, NH_3_ concentrations (*P* = 0.03), ${\text{NO}}_{2}^{-}$ / ${\text{NO}}_{3}^{-}$ concentrations (*P* = 0.03), and redox potential (*P* = 0.03) were significant in determining the distribution of MAGs (permutation test: *P* < 0.05). We further observed that the sites with the greatest diversity (L/C sites) were also those with the greatest redox potential, and O_2_ and ${\text{NO}}_{3}^{-}$/${\text{NO}}_{2}^{-}$ concentrations. Sites with the lowest microbial diversity (H sites), contained greater NH_3_ and ${\text{SO}}_{4-}^{2-}$ concentrations, and lower redox potential relative to the C and L sites. In addition to gradients shaped by the interplay between microbial metabolism and local geochemical constraints, the physical disturbances associated with high sedimentation rates also likely contributed to the loss of microbial diversity; however, we cannot quantify the relative importance of each of these processes here.

###  Contrasting Low vs. High Runoff Transects Also Revealed a Loss of Functional Potential

To assess the functional implications of this decrease of biodiversity, we assigned metabolic functions and pathways to proteins in each MAG. We focused on genes and pathways involved in key elements, targeting carbon, nitrogen, and sulfur cycling (Supplementary Figures 10, 11). Only the most abundant genomes per site were reported within each phylum (Supplementary Figure 12), allowing us to compute the proportions of functions and pathways in each of the 25 phyla present in reconstructed MAGs across the hydrological regimes (Supplementary Figures 13, 14). Their hierarchical clustering (Supplementary Figure 13) led to a picture consistent with the ones derived from both geochemical (Figure 1) and taxonomic abundances (Figure 4A). Indeed, the two transects were again clearly separated [clustering pattern (((L,C),S),H); Figure 4B, inset], and fitting linear models regressing function/pathway proportions against sites showed that, again, most of these slopes were negative (binomial test: *P* = 0.0010). Forcing the same site ordering as for the taxonomic abundances (L→C→H→S as in Figure 4A, inset) led to similar results (binomial test: *P* = 7.8 × 10^−5^), demonstrating a significant decrease in metabolic diversity when going from the L to the H transect.

More specifically, we found that marker genes whose product is implicated in carbon and sulfur metabolisms significantly decreased when going from the L to H, while nitrogen metabolism was unaffected (Supplementary Table 3, see Supplementary Text for details). When considering the individual functions present or absent across the transects, we noted that most oxidative pathways (CO, methane, formaldehyde, sulfide, sulfite) appeared less common in the H transect (Supplementary Figure 10), corresponding to lower oxygen concentrations and constraints on aerobic metabolism. Furthermore, while most carbon fixation processes were shared between the two transects, carbon oxidation and reduction reactions regulated through Wood-Ljungdahl pathway were only observed in the H transect, where sedimentary conditions were anoxic throughout the first 5 cm (see Figure 4 in St.Pierre et al., 2019b), consistent with a more reductive environment.

## Discussion

Even if Arctic microbial communities are changing rapidly (Hultman et al., 2015), there is still a dearth of long-term time series observations. As a first step toward addressing this point, we used Lake Hazen's spatial geochemical heterogeneity to evaluate the structural and functional response of lake sediment microbial communities to varying runoff, already shown to increase in this warming High Arctic environment (Lehnherr et al., 2018). Such an approach can reasonably be interpreted from the lens of a space-for-time design, which assumes that spatial and temporal variations are not only equivalent (Blois et al., 2013; Lester et al., 2014), but also stationary (Damgaard, 2019). Whether this latter condition is met cannot be known, but in the absence of any time-series documenting the effect of climate change on lake sediment microbial communities in the High Arctic, the space-for-time design becomes a convenience, if not a necessity (Pickett, 1989).

Using metagenomics along two transects experiencing heterogeneous runoff conditions, we presented evidence that climate change, as it drives increasing runoff and sediment loading to glacial lakes, will likely lead to a decrease in both diversity and functional potential of the dominant microbial communities residing in lake sediments. Note that we specifically focused here on the dominant microbes, that is those for which we could reconstruct the MAGs, in order to (i) have a phylogenetic placement of the corresponding organisms based on a large number of marker genes (Figure 2), rather than partial 16S rRNA gene sequences as usually done (Ruuskanen et al., 2018), and (ii) be able to predict almost complete functional pathways for each of these organisms to test the impact of a change of runoff (Figure 4), rather than inferring function from taxonomic affiliation (Ruuskanen et al., 2018). On the other hand, by focusing on dominant microbes, we lose taxonomic depth, and may miss key ecological and geochemical roles played by the “rare biosphere” (Lynch and Neufeld, 2015), and its associated shifts in the context of a warming climate.

Such a decrease in taxonomic and functional diversity may not be unique to Lake Hazen, where rising temperatures have resulted in increasing glacial melt and associated runoff. Although such a pattern has not been observed in other regions of the globe where runoff is predicted to decrease (Huss and Hock, 2018; St.Pierre et al., 2019b), our finding are likely to apply to other, smaller, glacierized watersheds typical of high latitudes or altitudes. Indeed, at least in the Arctic, freshwater discharge is broadly expected to increase with increasing temperatures and precipitation loadings (Peterson et al., 2002; Rawlins et al., 2010; Bring et al., 2016). It would thus be immensely valuable to conduct similar studies, replicating where appropriate a similar space-for-time design, at other lakes throughout the world. Additional sampling efforts should carefully consider the spatial heterogeneity of runoff regimes leading to divergent sedimentation rates (Supplementary Table 3), limiting our ability to make temporal predictions.

The hierarchical clustering showed that diversity at the phylum level generally decreased from the L to the H sites (Figure 4), and that the L and the C sites clustered together. This finding is unsurprising, as the L and C sites are neither distinct in terms chemical (Figure 1B) or taxonomic composition (Supplementary Figures 5, 6). Yet as the C sites are characterized by negligible runoff, this may suggest that either the L→C connection is not solely due to runoff, or that receiving low runoff temporarily increases taxonomic diversity. This observation that environments of low or intermediate disturbances can lead to maximum diversity is quite common and may provide a possible explanation for the ecological similarity between these two spatially separated sties (Gibbons et al., 2016).

Despite lacking geochemical measurements for the soil samples, we found that the microbial communities in the sediments at the high runoff sites clustered most frequently with those in the soil sites (Figure 4—see hierarchical clustering to the left of inset heatmaps), highlighting a potential connection between terrestrial and aquatic sediment communities as a function of the runoff volume, consistent with previous findings (Ruiz-González et al., 2015; Comte et al., 2018). In the High Arctic, glacial rivers travel across poorly consolidated landscapes with little vegetation, thus facilitating the rapid erosion and transport of materials, including soils. Glacial rivers in the High Arctic are therefore extremely turbid and discharge large loads of suspended materials and nutrients into Lake Hazen (St.Pierre et al., 2019a,b). Because of the large effect that these rivers have on the chemistry of downstream aquatic ecosystems, we would expect increased runoff to the aquatic ecosystems to alter microbial community structure (Le et al., 2016). Some of these structural changes may then alter the functional capacity to metabolize carbon, nitrogen, sulfur compounds, and process toxins such as metals and antibiotics (Supplementary Figure 10). A more experimentally-driven approach, based for instance on *in situ* incubation and geochemical tracers, would have been necessary to quantify such an interplay between microbial metabolism and geochemical features. Yet, as sediments and nutrients are mostly deposited during the summer melt months, it can be expected that lake sediments record microbe-driven seasonal changes in their geochemistry. Indeed, high glacial runoff is known to bring dense, oxygenated river waters with OC directly to the bottom of the lake (St.Pierre et al., 2019b), stimulating aerobic microbial activity. As a result, the geochemistry recorded along the high runoff transect may first reflect a period of greater microbial metabolism, which may actually exceed those in temperate systems (Probst et al., 2018), eventually followed by low oxygen, low redox, and high NH_3_ conditions observed here (Figure 1) as oxygen is depleted and anaerobic metabolisms allowed to proceed.

At a larger temporal scale, a key question that arises from these results is how changes in hydrological regimes will alter the evolutionary dynamics of microbial communities in lake sediments. Niche differentiation, where the coexistence of ecological opportunities can facilitate species diversification, may explain why sediments along the low runoff transect hosts a more diverse microbial community than sediments along the high runoff transect (Cordero and Polz, 2014). Presently, climate change is predicted to increase runoff in this High Arctic environment (Lehnherr et al., 2018), and we found evidence suggesting that the increased runoff decreased diversity at the phylum level (Figure 4). This can be expected to disrupt niche differentiation, and hence to reduce the overall and long-term metabolic capacity in lake sediments. It is currently hard to predict the future microbial ecology of these systems. On the one hand, climate change may diminish species diversification, and lead to highly specialized microbial communities adapted to a uniform ecological niche characterized by low oxygen, low redox, and high NH_3_ concentrations. On the other hand, the seasonal and rapid changes in redox conditions, predicted to follow the strong but punctual input of oxygen and nutrients during springtime may allow for the development of a short-lived community that eluded our sampling and analysis.

The rapid changes that affect Lake Hazen's watershed in response to climate warming were already known to directly alter its hydrological regime. Here we further provide evidence that a combination of increasing runoff and changing geochemical conditions are associated with the reduced diversity and metabolic potential of its dominant microbial communities. While longitudinal studies are needed to confirm these patterns, it is still unclear how such losses in biodiversity and metabolic potential in Arctic ecosystems will impact key biogeochemical cycles, potentially creating feedback loops of uncertain direction and magnitude.

## Materials and Methods

###  Sample Collection and Processing

Sediment and soil cores were collected from Lake Hazen (82°N, 71°W: Figure 1A), located within Quttinirpaaq National Park, on northern Ellesmere Island, Nunavut. Sampling took place between May 10 and June 10, 2017, when the lake was still completely ice-covered and prior to the onset of glacial melt within the watershed (Supplementary Table 4). Between late June and the end of August, meltwaters flow from the outlet glaciers along the northwestern shoreline through poorly consolidated river valleys, depositing sediments at the bottom of Lake Hazen. Sediment cores were sampled along two transects, the H1/H2 and L1/L2 sites, representing areas of the lake influenced by large (high runoff) and small (low runoff) rivers, respectively (Supplementary Table 5, adapted from St.Pierre et al., 2019b). The lake then drains via the Ruggles River along its southeastern shoreline (C site). The surrounding glacial rivers deliver different amounts of sediments, nutrients, and organic carbon unevenly to the lake as a consequence of heterogeneous sedimentation rates (Supplementary Table 6, adapted from St.Pierre et al., 2019b). More specifically, the top 5 cm of sediments from the deeper low (L2) and high (H2) runoff sites represented depositional periods of 30 years (1987–2017) and 6 years (2011–2017), respectively.

Samples were collected along two transects and can be separated into three hydrological regimes by seasonal runoff volume: low (L transect), high (H transect), and negligible runoff (C sites) summarized in Supplementary Table 5. Contamination of samples was minimized by wearing non-powdered latex gloves during sample handling and sterilizing all equipment with 10% bleach and 90% ethanol before sample collection. Sediment cores approximately 30 cm in length were collected with an UWITEC (Mondsee, Austria) gravity corer from five locations: C (overlying water depth: 50 m) far from the direct influence of glacial inflows serving as a control site; L1 (water depth: 50 m) and L2 (water depth: 251 m), at variable distances from a small glacial inflow (Blister Creek, <0.001 km^3^ in summer 2016); and, H1 (water depth: 21 m) and H2 (water depth: 253 m), located adjacent to several larger glacial inflows (i.e., the Abbé River, 0.015 km^3^ and Snow Goose, 0.006 km^3^ in 2016). Note that bathymetry changes quickly on the northern side of Lake Hazen, making it challenging to match the shallow sites (L1 and H1) when coring through the ice-covered lake. The glacial inflow measurements were included in a previous study (St.Pierre et al., 2019b), and are summarized in Supplementary Table 5. The soil samples (S sites) were collected from three sites in the dried streambeds of the tributaries, on the northern shore between the two transects. At each site, for both sediments and soil, five cores were sampled, ~3 m apart for the sediment cores, and approximately ~1 m apart to account for site heterogeneity.

Microprofiling data were previously described (St.Pierre et al., 2019b). Briefly, for sediment core, one of the five cores was used for microprofiling of oxygen (O_2_), redox and pH, as well as one core for porewater chemistry and loss on ignition (see Ruuskanen et al., 2018, for details), and the remaining three cores were combined, prior to their genomic analysis, here again to account for site heterogeneity. For soil samples, three cores per site were collected for DNA analysis, but no additional cores were collected for chemical analyses. As we were mostly interested in the community composition through space, we combined the top 5 cm of sediment and 10 cm of soil for sample extraction and subsequent sequencing (below 10 cm, the ground was frozen and could not be penetrated safely by our corer). Critically, surface vegetation was scrubbed off if present and not included in the extraction vials. This was done to limit any plant DNA being captured in the extraction process. Any remaining length of cores that were used for DNA analysis were discarded. These uppermost layers in the sediment correspond to both the most recent sediment deposition dates and the region of greatest microbial activity (Haglund et al., 2003). Based on previous findings (St.Pierre et al., 2019b), sediment below 10 cm in depth can date back as early as 2009 in the high runoff sites, or as late as 1900 in low runoff sites (Supplementary Table 6). Although the age of the sediment at a given sample depth can vary, we were most concerned with how the microbial community in the most active depths responded to changing environmental conditions as a result of increased runoff and sediment delivery. The top of each core was sectioned and placed into Whirlpack bags. These slices were homogenized manually inside of the bags and stored in a −20°C freezer until shipment back to the University of Ottawa where they were then stored at −80° C. Soil samples were transferred into falcon tubes, homogenized, and stored as described above for the lake sediment samples.

Samples were thawed overnight and 250–400 mg (wet weight; Supplementary Table 7) were then washed in a sterile salt buffer (10 mM EDTA, 50 mM Tris-HCl, 50 mM Na_2_ HPO_4_ 7H_2_O at pH 8.0) to remove PCR inhibitors (Zhou et al., 1996; Poulain et al., 2015). All sample handling was conducted in a stainless-steel laminar flow hood (HEPA 100) treated with UVC radiation and bleach before use. DNA extractions were performed using the DNeasy PowerSoil Kit (MO BIO Laboratories Inc, Carlsbad, CA, USA), following the kit guidelines, except that the final elution volume was 30 μl instead of 100 μl. DNA integrity was validated with a NanoDrop Spectrometer and PCR combined with electrophoresis of the Glutamine synthetase gene (*glnA*) as this gene is ubiquitous across microbial life (Supplementary Figure 15, Supplementary Table 8). Adequate DNA concentrations for sequencing were reached by combining triplicate extractions for a total volume of 45 μl and a concentration ≥ 50 ng/μl (Supplementary Table 7). Positive and negative controls were used to verify the integrity of the PCR amplification of *glnA* (see also Ruuskanen et al., 2020). Two kit extraction blanks contained no trace of DNA and were not sequenced.

###  Chemical Analyses

Redox potential, pH, and dissolved O_2_ concentration profiles were measured at 100 μm intervals on one of the cores within an hour of collection, using 100 μm Unisense (Aarhus, Denmark) glass microsensors connected to a Unisense Field Multimeter (OX-100, pH-100 coupled to REF-RM, RD-100 coupled to REF-RM). All sensors were calibrated immediately before the profiles were measured using the standard calibration procedures outlined in the individual sensor manuals provided by the manufacturer. A 5 mm reference electrode (Ag-AgCl; Ref-RM) was coupled with both the pH and redox potential sensors and kept in the water overlying the core during profiling. All profiles were begun at ~1 cm above the sediment surface, which was approximately uniform at all sites. At each step, the probes equilibrated for 10 s before taking triplicate measurements, which were averaged to produce the profiles. A single profile was conducted on each core to measure the natural *in situ* biogeochemical gradients present at each site. We note that redox profiles should be interpreted as relative rather than absolute differences (Boudreau and Jorgensen, 2001, p. 180–210). Sediment porewater was extracted following centrifugation at 4,000 rpm. The supernatant was then filtered through 0.45 μm cellulose acetate filters into 15 ml tubes, and were frozen until analysis. Concentrations of nitrates and nitrites (${\text{NO}}_{2}^{-}$ + ${\text{NO}}_{3}^{-}$), and ammonia (NH_3_), chloride (Cl^−^) were measured in the sediment porewater using a Lachat QuickChem 8500 FIA Ion Analyzer, while total dissolved phosphorus (TDP) and ${\text{SO}}_{4-}^{2-}$ were measured in the sediment porewater using an ion chromatograph at the Biogeochemical Analytical Service Laboratory (Department of Biological Sciences, University of Alberta). However, TDP was removed from data analysis because insufficient porewater was collected to measure TDP at site C. The centrifuged sediments were retained and percentage per dry weight (% d.w.) of calcium carbonate (CaCO_3_) and organic carbon (OC) were estimated through loss on ignition (Heiri et al., 2001).

The chemical features of the top 5 cm of the sediment cores were derived from measurements performed at 1 cm intervals throughout the cores, and were reported in St.Pierre et al. (2019b) as part of a larger study on the Lake Hazen watershed, which did not investigate microbial communities in the lake. The geochemical properties of each sediment site were summarized using a Principal Component Analysis (PCA) that was scaled to unit variance and projections were clustered using Partitioning Around Medoids (Maechler et al., 2019). The appropriate number of clusters was determined from silhouettes with the R package hopach (van der Laan and Pollard, 2003). The Dunn test R package (Dinno, 2017) was used to compare samples, controlling for multiple comparisons with the Benjamini-Hochberg adjustment.

###  Sequencing and Data Processing

Metagenomic libraries were prepared and sequenced by Genome Quebec on an Illumina HiSeq 2500 platform (Illumina, San Diego, CA, USA; Supplementary Figure 16) on a paired-end 125 bp configuration using Illumina TruSeq LT adapters (read 1: AGATCGGAAGAGCACACGTCTGAACTCCAGTCAC, and read 2: AGATCGGAAGAGCGTCGTGTAGGGAAAGAGTGT). The DNA from the eight sites (five sediment and three soil) was sequenced with two samples per HiSeq lane, generating a minimum of 125 million reads per sample, which amounted to over 150 GB of sequencing data. Read count summaries were tracked throughout each step of the pipeline for quality control (Supplementary Figure 17). Low quality reads, adapters, unpaired reads, and low quality bases at the ends of reads were removed to generate quality controlled reads with Trimmomatic (v0.36) (Bolger et al., 2014) using the following arguments: phred33, ILLUMINACLIP:TruSeq3-PE-2.fa:3:26:1 0, LEADING:3TRAILING:3,SLIDINGWINDOW:4:20,MINLEN:36, CROP:120,HEADCROP:20,AVGQUAL:20. Then, FASTQC (v0.11.8) (https://www.bioinformatics.babraham.ac.uk/projects/fastqc/) was used to confirm that the Illumina adapters were removed and that trimmed sequence lengths were at least 90 bp in length with a Phred score of at least 33.

###  Reconstruction of Environmental Genomes and Annotation

To reconstruct environmental genomes, metagenomic quality-controlled reads from all samples were coassembled using Megahit (Li et al., 2015) software with a k-mer size of 31 and meta-large setting (see Supplementary Table 1 for additional summary statistics). EukRep (West et al., 2018) was used to remove any eukaryotic DNA from the contigs prior to the formation of an Anvio (v5) (Eren et al., 2015) contig database. The contig database was generated by removing contigs under 1000 bp, and gene prediction was performed in the Anvio environment. Sequence coverage information was determined for each assembled scaffold by mapping reads from each sample to the assembled contig database using Bowtie2 (Langmead and Salzberg, 2012) with default settings. The resulting SAM files were sorted and converted to BAM files using samtools (v0.1.19) (Li et al., 2009). Each BAM file was prepared for Anvio using the anvi-init-bam and contig database generated using anvi-gen-contigs-database. The contig database and BAM mapping files were further used as input for anvi-profile, which generated individual sample profiles for each contig over the minimum length of 2,500 bp. These profiles were then combined using anvi-merge and summary statistics for abundance and coverage were generated with anvi-summarize. Automated binning was performed using CONCOCT (Alneberg et al., 2014). Scaffolds were binned on the basis of GC content and differential coverage abundance patterns across all eight samples. Manual refinement was done using Anvio's refine option, where contigs were manually removed from bins on the basis of GC content, differential abundance in samples, and taxonomy of contigs assigned by Kaiju (Menzel et al., 2016, Supplementary Table 2). Kaiju was used to classify taxonomy of the assembled contigs with anvi-import-taxonomy-for-genes and aided in the manual refinement process. Open reading frames were predicted with Prodigal (v2.6.3) (Hyatt et al., 2010). Anvio's custom Hidden Markov Models were run, along with NCBIs COG (Tatusov et al., 2003) annotation to identify protein-coding genes. PFAM (Finn et al., 2015), TIGRFAM (Haft et al., 2003), GO terms (Ashburner et al., 2000), KEGG enzymes and pathways (Kanehisa et al., 2015), and Metacyc pathways (Caspi et al., 2007) were predicted with Interproscan (v5) (Jones et al., 2014). These annotations were then combined with the Anvio database with anvi-import-functions.

Genome completeness and contamination were evaluated on the presence of a core set of genes using CheckM (v1.0.5) lineage_wf (Supplementary Table 2, Supplementary Figure 18; Parks et al., 2015). Only the 300 genomes that satisfied the quality control cutoffs of at least 50% complete and with <10% contamination were further analyzed—meeting the Minimum Information about metagenome-assembled genome (MIMAG) of bacteria and archaea for medium or high-quality genomes (Bowers et al., 2017). All recovered genomes were used to calculate an average amino acid identity across all genomes using compareM (v0.0.23, function aai_wf; https://github.com/dparks1134/CompareM; Parks et al., 2017). CheckM was used again to identify contigs that were not contained in any of the 300 high-quality genomes, that is those whose size ranges from 1,000 to 2,500 bp. As an attempt to “rescue” these unbinned contigs, an alternative binning algorithm MaxBin (v2.0) (Wu et al., 2015) was employed. An additional 481 genomes were recovered, but were not included in further analysis as only 21 genomes were of average completion >65% (Supplementary Data 1: github.com/colbyga/hazen_metagenome_publication/blob/master/Supplemental_Data_1_maxbin2_unbinned_contigs_summary.csv).

###  Phylogenetic Placement of the MAGs

Phylogenetic analyses were performed using two different sets of marker genes from the Genome Taxonomy Database (GTDB): one for bacteria (120 marker genes) and one for archaea (122 marker genes), as previously been used to assign taxonomy to MAGs (Parks et al., 2018). The marker genes were extracted from each genome by matching Pfam (v31) (Finn et al., 2015) and TIGRFAM (v15.0) (Haft et al., 2003) annotations from GTDB (v86) (Parks et al., 2018). Marker genes from each of the 300 genomes were translated using the R package seqinr (Charif and Lobry, 2007), selecting the genetic code that returned no in-frame stop codon. As some genomes had multiple copies of a marker gene, duplicated copies were filtered out by keeping the most complete sequence. Marker genes that were missing from some genomes were replaced by indel (gap) characters, and their concatenated sequences were added those from the reference GTDB sequences. MUSCLE (v3.8.31) (Edgar, 2004) was employed to construct the alignment in R (v 3.5.1) (R Development Core Team, 2008). Archaeal sequences were removed from the bacterial alignment on the basis of results from CheckM (Parks et al., 2015) and independently verified using a custom list of archaea specific marker genes. Alignments were then refined using trimAI (Capella-Gutiérrez et al., 2009) and the -gappyout parameter. FastTree2 (Price et al., 2010), recompiled with double precision to resolve short branch lengths, was used to infer maximum likelihood phylogenetic trees from protein sequence alignments under the WAG +Γ model (Whelan and Goldman, 2001; Aris-Brosou and Rodrigue, 2012, 2019). The archaeal tree was rooted with Euryarchaeota and the bacterial tree was rooted with Patescibacteria using the R package APE (Paradis et al., 2004). Trees were visualized and colored by phylum with the R package ggtree (Yu et al., 2017).

###  Community Composition of the MAGs

To determine the relative abundance of each genome in the eight samples, sample-specific genome abundances were normalized by sequencing depth [(reads mapped to a genome)/(total number of reads mapped)], making comparisons across samples possible. Genome abundances were generated using the CheckM profile function (Parks et al., 2015). To determine the average abundance of major taxonomic groups across sites (determined by the phylogenetic analysis described above), the abundances for genomes from the same taxonomic group were summed and visualized using the R package phyloseq (McMurdie and Holmes, 2013). These same abundance values were the basis for a community composition analysis. The *t*-SNE plots were constructed by assigning each genome to a site based on where it was most abundant using the R package Rtsne (Krijthe et al., 2018). All these analyses were made at the phylum level, unless otherwise stated.

###  Metabolic Potential of the MAGs

To analyze functional marker genes in the metagenomes, we used a custom database of reference proteins sequences (COG, PFAM, TIGRFAM, KEGG) based on the marker genes used in other studies (Anantharaman et al., 2016; Dombrowski et al., 2018, Supplementary Data Files on GitHub). Pathways were also predicted using MinPath (Ye and Doak, 2009) to map all identified KEGG enzymes to the most parsimonious MetaCyc pathways (Caspi et al., 2007). As these MAGs were incomplete, some genes in pathways may be absent. MinPath presented only parsimonious pathways represented by multiple genes. As most genomes were present even at low abundances across all sites, a cut-off value of ≤0.25 (on a −log_10_ scale) was set for a genome to be included in the functional analyses at any site, so that only the most abundant genomes for each site were considered. We aggregated marker genes and pathways by function, summarizing the results by phyla, except for Proteobacteria that were separated by class. We further grouped all taxa together at each site to test for significant differences in major nutrient cycling processes (carbon, nitrogen, and sulfur) among sites using a hierarchical clustering; significance was derived from the Approximately Unbiased bootstrap (Suzuki and Shimodaira, 2006) and Fisher's exact test.

## Data Availability Statement

The datasets presented in this study can be found in online repositories. The names of the repository/repositories and accession number(s) can be found below: https://www.ncbi.nlm.nih.gov/bioproject/PRJNA556841.

## Author Contributions

GC and VS performed sampling, whereas GC and MR conducted laboratory analyses. GC, MR, and SA-B performed data analyses. GC, SA-B, VS, and AP designed the study and wrote the manuscript. KS and VS conducted the microsensor profiles and porewater extractions. GC, SA-B, AP, MR, KS, and VS reviewed the manuscript. All authors contributed to the article and approved the submitted version.

## Conflict of Interest

The authors declare that the research was conducted in the absence of any commercial or financial relationships that could be construed as a potential conflict of interest.
