# Supplementary material for: Warming Climate Is Reducing the Diversity of Dominant Microbes in the Largest High Arctic Lake
Source: Front Microbiol. 2020 Oct 7;11:561194. doi: 10.3389/fmicb.2020.561194 (PMC7579425; doi:10.3389/fmicb.2020.561194)
Supplement: Supplementary file 1 [file Data_Sheet_1.PDF]

Supplementary Information

Warming climate is reducing the diversity of dominant microbes in the largest High Arctic lake

Graham A. Colby[1], Matti O. Ruuskanen[1], Kyra A. St. Pierre[2], Vincent L. St. Louis[2], Alexandre J. Poulain[1], Stéphane Aris-Brosou[1,3]

[1]Department of Biology, University of Ottawa, Ottawa, ON K1N 6N5, Canada. [2]University of Alberta, Department of Biological Sciences, Edmonton, AB T6G 2E9, Canada. [3]Department of Mathematics and Statistics, University of Ottawa, Ottawa, ON K1N 6N5, Canada. Correspondence and requests for materials should be addressed to S.A.B (email: `sarisbro@uottawa.ca`)

Supplementary Text

Nutrient cycles affected when transitioning from low to high. Overall, markers of carbon and sulfur metabolism significantly decreased when transitioning from the L to H sites, even if nitrogen metabolism was not (Supplementary Table 3). Most carbon pathways, such as carbon fixation through the Calvin-Benson-Bassham (CBB) pathway, as well as the capacity for simple carbon metabolism, were shared across all runoff regimes. In contrast, carbon oxidation and reduction reactions regulated through Wood-Ljungdahl pathway were only observed in the H sites, where sedimentary conditions were anoxic throughout the first 5 cm (see Figure 4 in [10]). Here, Spirochaetota were likely performing anaerobic respiration and carbon fixation producing acetate as an end product. Methanogenesis pathways were present across all sites, but notably, methane oxidation pathways were absent from high runoff sites, where oxygen is limited.

Greater concentrations of ammonia in the high runoff regimes may suggest that N-containing organic matter was mineralized through ammonification (Supplementary Figures 1 and [10]). In the high runoff regime, there was both an absence of nitrification and a greater presence of markers for ammonia assimilation. Markers for dissimilarity nitrate reduction (DNRA) were present in multiple genomes across all runoff regimes (Supplementary Figure 10). In contrast, urease markers were found more abundantly in low runoff regimes, where ammonia concentration was lower (see Figure 4 in [10]). The functional ability of microbes to cycle sulfur between oxidized and reduced forms was significantly different between the high and low runoff regimes (Supplementary Table3). In the high runoff regime, Gammaproteobactiera were the only organisms with the metabolic capacity to expansively utilize sulfur, performing sulphide oxidation and thiosulfate reduction. Whereas, sulphate reduction was predominantly found in the soil, control, and low runoff regimes.

Aside from nutrient cycling, we also assessed the capacity of microbial communities to process metals and antibiotics. Metal resistance and cycling was mostly ubiquitous throughout all of the sites, regardless of runoff. Methyl mercury production, identified by the presence of both hgcA and hgcB genes [1], was only implicated in the high runoff sites, in Spirocheatoa and Chloroflexoata. However, genes conferring mercury resistance involved in the conversion of inorganic Hg^{II} to the less toxic Hg^0 – were evenly distributed throughout the sites. There was a broad presence of metal tolerance that was indicated by genetic determinants related to heavy metal resistance of cadmium, cobalt, copper, lead and zinc (Supplementary Figure 10). Furthermore, antimicrobial resistance genes specifically β-lactamases, were ubiquitous across all genomes at all sites. Finally, we found that while amino acids were readily synthesized and degraded by most organisms (Supplementary Figure 10), the metabolic potential to degrade of polycyclic aromatic compounds appeared to be least prevalent in the high runoff sites.

Sites clustered by runoff regime. To identify potential drivers of this reduction in diversity when going from the L to the H transects, we bi-clustered genomes by their normalized abundances (on a $-\log_{10}$ scale to reduce skew) and by sample site (Supplementary Figure 13), and found that sites clustered following a similar pattern to geochemical features (see Figure 1B), with H sites grouping separately from L sites (Figure 3A). The normalized abundances of MAGs showed no strong phylogeographic pattern, in that we did not observe an assemblage of MAGs solely representative of a given site (Supplementary Figure 14). In spite of this absence of phylogeographic pattern, the tanglegram suggests that the beta diversity of highly abundant MAGs in the L/C sites was greater than at the H sites (see the distribution of green lines connecting the phylogenetic and clustered trees in Supplementary Table14). This difference in diversity between samples is further supported by an NDMS ordination (Supplementary Figure 9), a PERMANOVA test on a PCoA ordination (Supplementary Figure 5) and a DPCoA ordination (Supplementary Figure 6).

Production of ordinations. Ordinations are a commonly used method to condense multivariate data, and it has conventionally applied to OTU tables that record the number of reads per sample that belong to a specific OTU. As a substitute for this OTU metric, we used the number of reads mapped to each MAG per sample, which is standard output from Anvio [2], to form the ordination matrix. This matrix of mapped reads per sample were input into phyloseq (ver. 1.26.0) [3] to produce abundance plots of all samples and all taxa (Figure 2 in main text and Supplementary Figure 4). Furthermore, phyloseq [3] was used to produce a NMDS biplot using only the top 20 most abundant taxa in the sediment samples (Supplementary Figure 9). For Supplementary Figure 9, only sediment sites were used in the ordination as chemical data was required to add environment vectors onto the ordinations using vegan (ver. 2.5.3) [4]. The tree structure used to create the phyloseq object was based on the combined archaeal and bacterial tree produced by checkM [5]. Significant chemical vectors shown in Supplementary Figure 9 in were marked in red using vegan's envifit function.

The PCoA plot (Supplementary Figure 5) and the DPCoA plot (Supplementary Figure 6) were produced using a similar approach described above, using the proportion of MAGs per sample to form a matrix. Supplementary Figure 5, was produced using vegan (ver. 2.5.3) [4] to plot the samples in an ordination space based on the distribution MAGs in each sample. The results from the PCoA plot (Supplementary Figure 5) indicate that there is a unique clustering (or diversity of MAGs) between the S, L, and H sites. The DPCoA biplot (Supplementary Figure 6) was produced with phyloseq (ver. 1.26.0) [3] and presents all 300 MAGs and relative placement of sampling sites separated onto two plots. The two DPCoA plots result from two trees unique GTDB trees [6], an archaeal and bacterial tree. The archaea DPCoA (Supplementary Figure 6B) contains only the 10 recovered archaeal MAGs, limiting the discrimination power and interpretation of diversity trends. However, the bacterial DPCoA (Supplementary Figure 6A) contains 290 MAGs

and strongly suggests a trend of increase diversity from S to H to L and C sites. On the bacterial DPCoA, there is a clear placement of L1, L2 and C sites in the "most diverse region" of the ordination containing the most unique phyla, the soil sites (H-Soil, C-Soil, L-Soil) cluster in a "lowest diversity region" containing only Actinobacteria and Gammaproteobacteria, and H1 and H2 sites reflect a diversity somewhere between the S and L sites. The placement of sample sites on the DPCoA (Supplementary Figure 6A) reflects the abundance of MAGs per site (Supplementary Figure 4).

Assessment of the impact of indels and missing data. To evaluate the impact of gaps and missing data on the phylogenetic placement of the archaeal and bacterial MAGs, we constructed new alignments based on the original ones – which we call the "full alignments" (there is one for archea, and one for bacteria; see Figure 2). From those, we constructed "half-gapped alignments," that were obtained with Gblocks [7], removing sites that contained $> 50\%$ of indels, and reconstructed the phylogenetics tree as in the main text. We then computed the Robinson-Foulds (RF) topological distance [8] between the full and the half-gapped trees. In order to gauge the significance of this distance, we rearranged the half-gapped tree by Subtree Pruning and Regrafting [9, SPR], a single rearrangement away from the input tree. We did not use bootstrapped trees for computational reasons (the estimation of the bacterial tree takes ~ 32 h). Because of the number of sequences in the bacterial tree, SPR rearrangements failed (integer overflow on a 64 bit computer); to alleviate this issue, we subsampled all trees to keep only 10% of the original taxa. For each subsampled tree, we computed the RF distance between the subsampled full tree and the subsampled rearranged half-gapped tree, did this for 1,000 replicates, and computed the P-value of observing RF distances that were greater than the observed distance. Because this P-value depends on the subsampling, we repeated this entire procedure 500 time, hence obtaining a distribution of P-values measuring the probability of observing a half-gapped tree that is more different than the full tree. The script developed for these analyses is available from `github.com/sarisbro/data/tree/master/miscel_scripts`.

The results show that each full (original) alignment contained sequences with a median of 15-20% of indels, but could contain up to 70% of missing data (Supplementary Figure 2A-3A). Missing data were reduced in the half-gapped data (Supplementary Figure 2B-3B), and led to unrooted topologies where the phylogenetic placements of the MAGs were not affected (Supplementary Figure 2C-D-3C-D), and where SPR-perturbed trees were significantly more distant from the full tree than the half-gapped tree (Supplementary Figure 2E-3E).

References

1. Parks JM, Johs A, Podar M, Bridou R, Hurt RA, Smith SD, et al. The genetic basis for bacterial mercury methylation. Science. 2013;339(6125):1332–1335.

2. Eren AM, Esen ÖC, Quince C, Vineis JH, Morrison HG, Sogin ML, et al. Anvio: an advanced analysis and visualization platform for omics data. PeerJ. 2015;3:1319.

3. McMurdie PJ, Holmes S. phyloseq: an R package for reproducible interactive analysis and graphics of microbiome census data. PloS one. 2013;8(4):e61217.

4. Oksanen J, Kindt R, Legendre P, O'Hara B, Stevens MHH, Oksanen MJ, et al. The vegan package. Community ecology package. 2007;10(631-637):719.

5. Parks DH, Imelfort M, Skennerton CT, Hugenholtz P, Tyson GW. CheckM: assessing the quality of microbial genomes recovered from isolates, single cells, and metagenomes. Genome research. 2015;25(9):1043–1055.

6. Parks DH, Chuvochina M, Waite DW, Rinke C, Skarshewski A, Chaumeil PA, et al. A standardized bacterial taxonomy based on genome phylogeny substantially revises the tree of life. Nature biotechnology. 2018;36:996–1004.

7. Talavera G, Castresana J. Improvement of phylogenies after removing divergent and ambiguously aligned blocks from protein sequence alignments. Systematic biology. 2007;56(4):564–577.

8. Robinson DF, Foulds LR. Comparison of phylogenetic trees. Mathematical biosciences. 1981;53(1-2):131–147.

9. Felsenstein J, Felenstein J. Inferring phylogenies. vol. 2. Sinauer associates Sunderland, MA; 2004.

10. St Pierre K, St Louis V, Lehnherr I, Schiff S, Muir D, Poulain A, et al. Contemporary limnology of the rapidly changing glacierized watershed of the world's largest High Arctic lake. Scientific reports. 2019;9(1):4447.

Supplementary figures

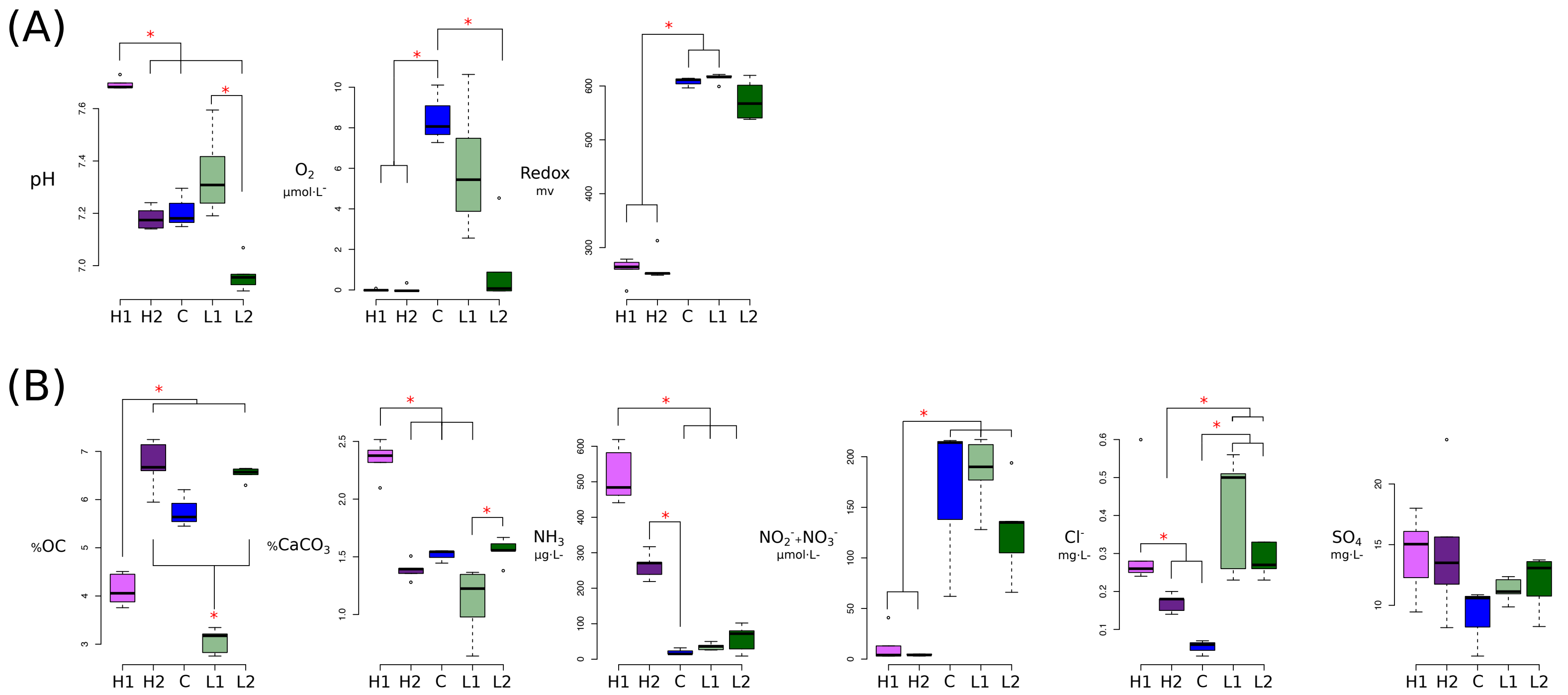

Supplementary Figure 1. Distribution of all chemical features for sediment sites. Includes chlorine and sulphate measurements absent from Figure 1. Branches and asterisks indicate significant differences between sites $P < 0.025$ (Dunn Test). If branches form a dichotomy or trichotomy, the interactions within that group is not significant. (A) Microprobe measurements collected at every 100 μm. (B) Porewater measurements collected from bulk 1 cm intervals. See [10] for measurement details.

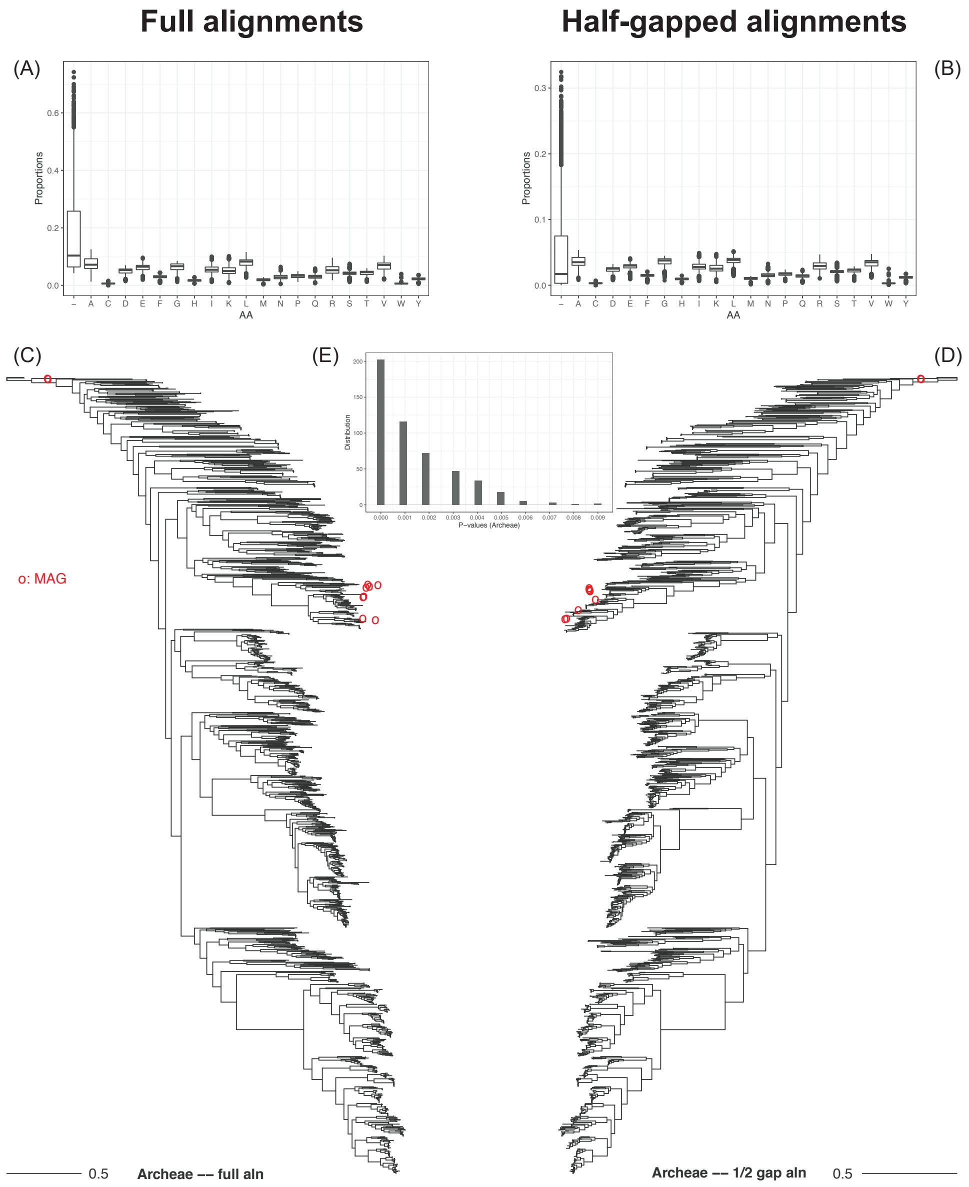

Supplementary Figure 2. Evaluation of the impact of gaps and missing data on the phylogenetic placement of the archaeal MAGs. The "full alignment" is the alignment used in Figure 2. The "half-gapped alignment" was obtained with Gblocks, removing sites that contained > 50% of indels. (A) Amino acid composition (indels as "−") of full alignment. (B) Amino acid composition (indels as "−") of half-gapped alignment. (C) Full tree. (D) Half-gapped tree. Both trees are arbitrarily rooted by a MAG, MAGs' placements indicated by red circles. (E) Distribution of P-values measuring the probability of observing a half-gapped tree that is more different than the full tree.

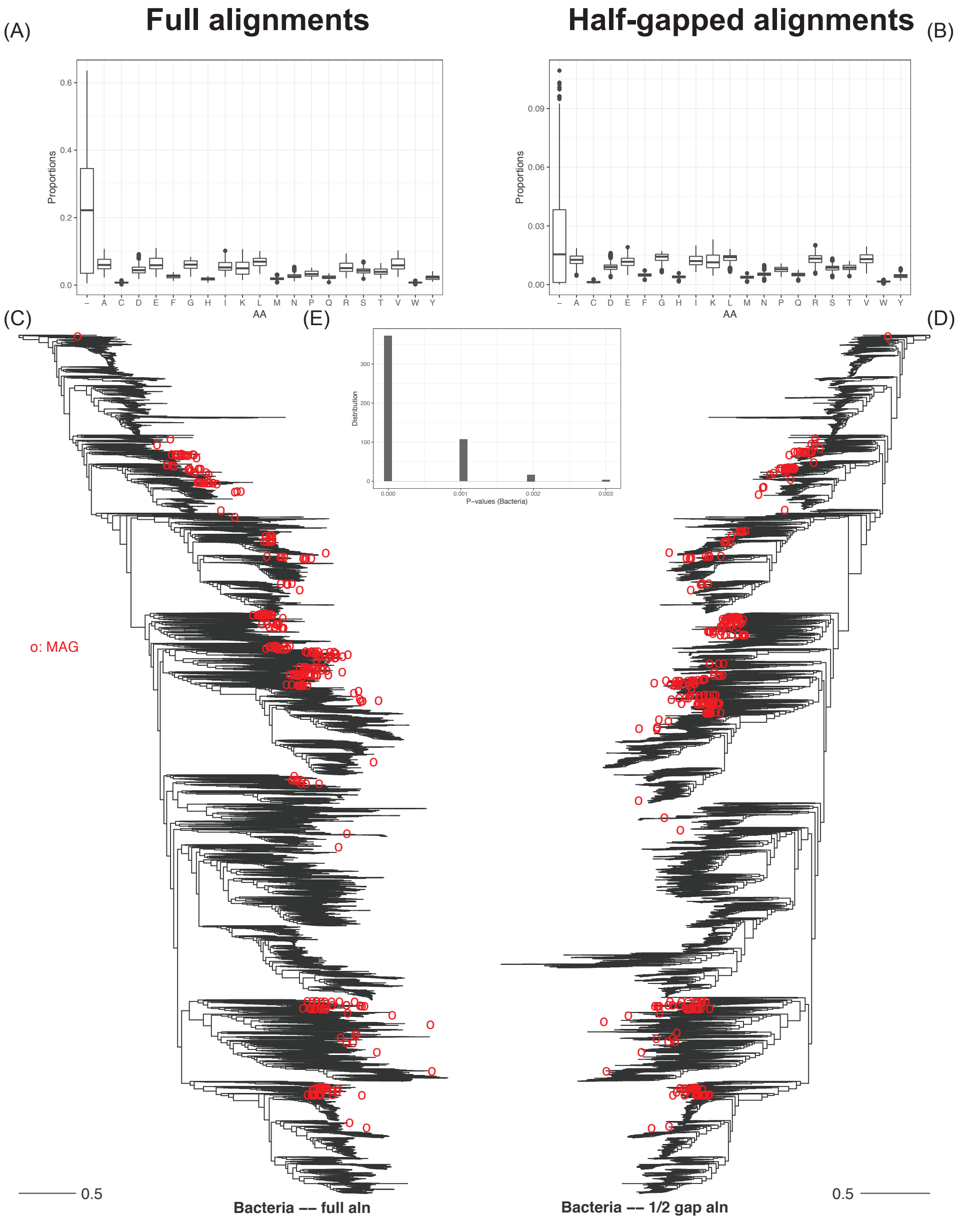

Supplementary Figure 3. Evaluation of the impact of gaps and missing data on the phylogenetic placement of the bacterial MAGs. The "full alignment" is the alignment used in Figure 2. The "half-gapped alignment" was obtained with Gblocks, removing sites that contained > 50% of indels. (A) Amino acid composition (indels as "–") of full alignment. (B) Amino acid composition (indels as "–") of half-gapped alignment. (C) Full tree. (D) Half-gapped tree. Both trees are arbitrarily rooted by a MAG, MAGs' placements indicated by red circles. (E) Distribution of P-values measuring the probability of observing a half-gapped tree that is more different than the full tree

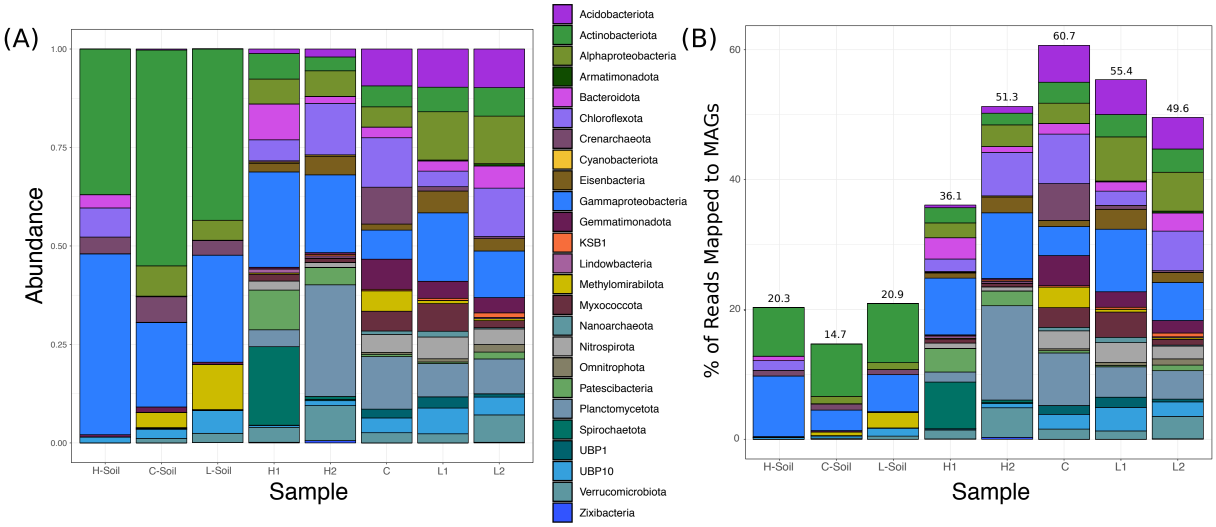

Supplementary Figure 4. Reconstructed genome abundance across sites. (A) Amount of reads mapped to the 300 high quality genomes for each sample normalized to 100%. Only reads that were mapped to genomes are shown and all unmapped reads have been excluded. (B) Amount of reads mapped to the 300 high quality genomes for each sample.

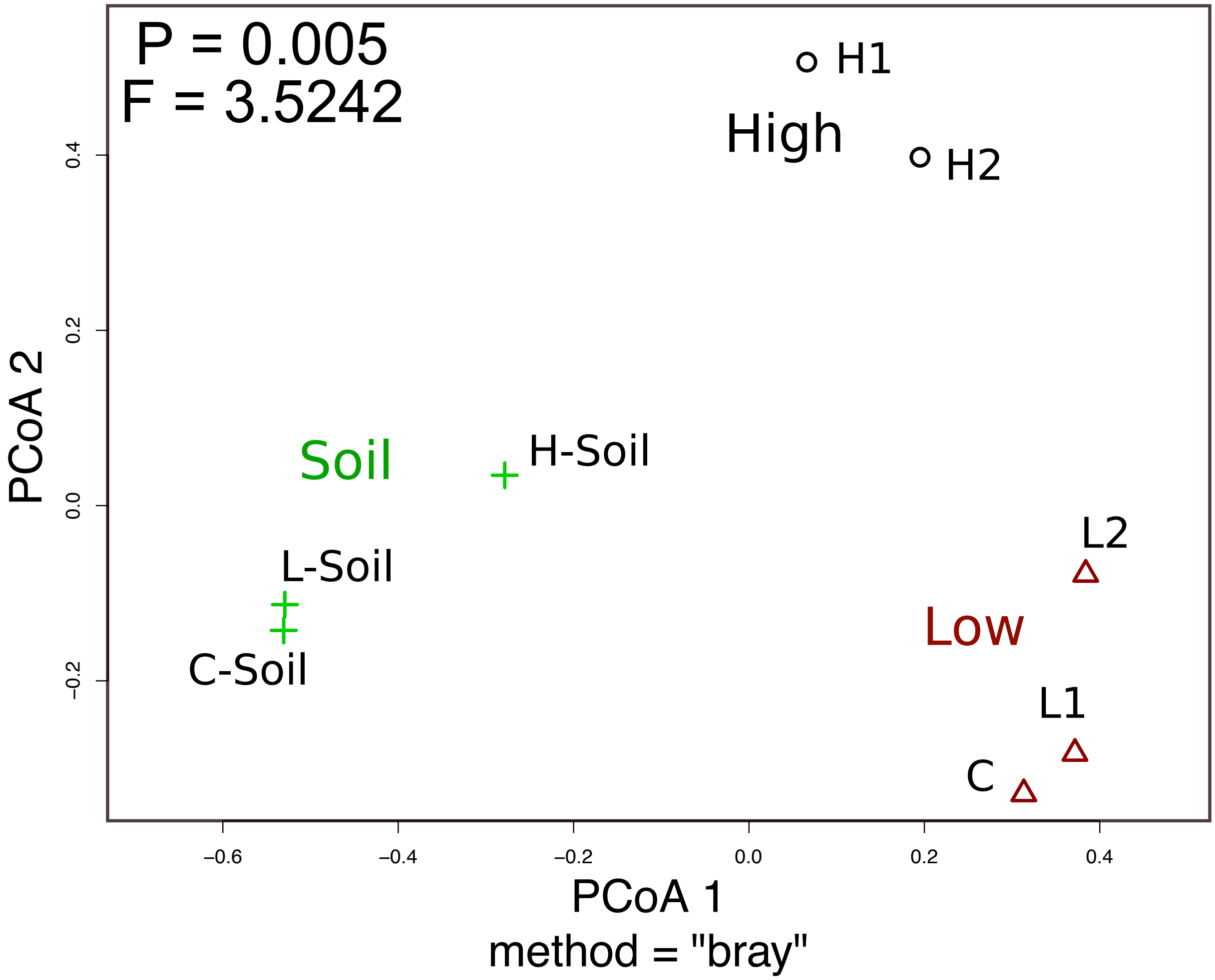

Supplementary Figure 5. Principal coordinate analysis (PCoA) on reconstructed genome abundances for all sites. Grouping the sites into soil (S), high (H), and low (L) runoff was significant (PERMANOVA test: $F = 3.52$, $P = 0.005$).

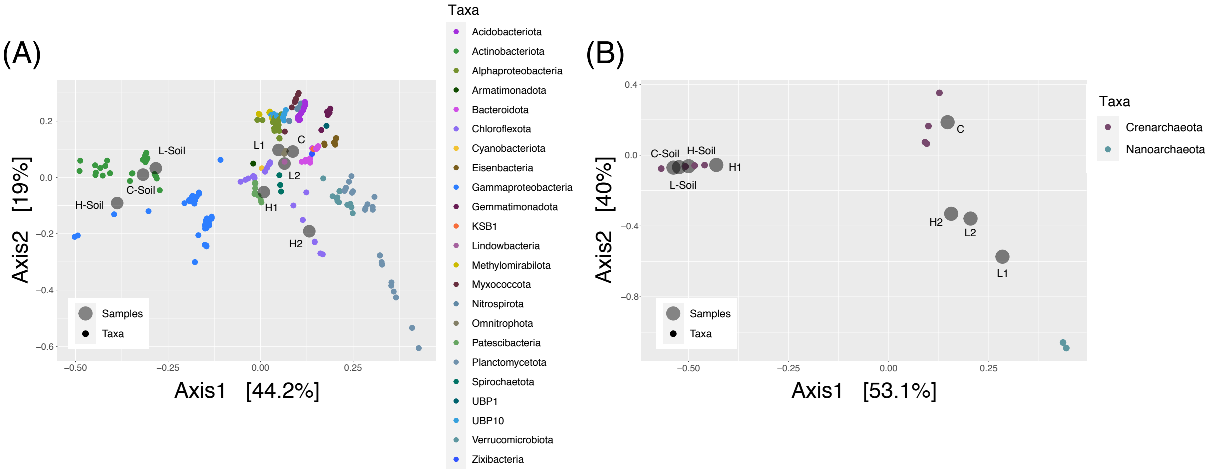

Supplementary Figure 6. Double Principal Coordinate Analysis (DPCoA) based on reconstructed genome abundances and phylogenetic trees. (A) DPCoA of bacterial genome abundances. (B) DPCoA of archaeal genome abundances. Phylum level genome classifications are shown (except for Proteobacteria which are grouped at the class level). Note that for the archaeal data, sample size is low with $n = 10$ MAGs, leading up to poor discrimination power.

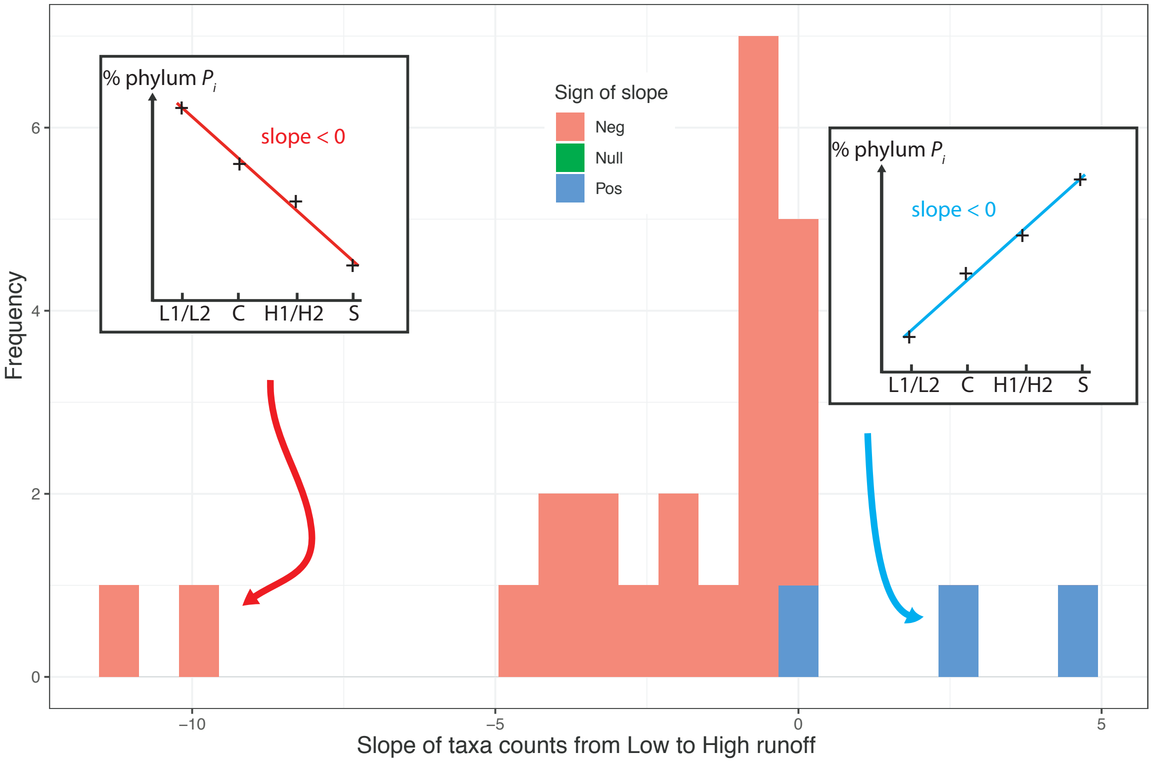

Supplementary Figure 7. How Figure 4 (main text) was constructed. Under the space-for-time design, we compare the proportion π_{P_i} of MAGs belonging to phylum P_i across four runoff levels ρ_i (from transects L1/L2, combined – the pre-warming situation – to transects H1/H2, combined – the post-warming situation – and S), and do that for the each of the 25 phyla represented by the MAGs we reconstructed ($i \in [1, 25]$). To test if the proportion of phylum P_i has increased through time (space), we fit a linear model: $\pi_{P_i} = a\rho_i + b + \epsilon$ (with $\epsilon \sim N(0, 1)$). This model is an ANOVA as the quantitative variable π_{P_i} is regressed against the factorial variable ρ_i. Because there is only one observation per factorial level (each runoff level has only one proportion of phylum P_i), the significance of ρ_i cannot be computed, but the slope $\hat{a}$ can nonetheless be estimated. A negative slope is tallied as such, and the same is done for positive slopes. A binomial test then assesses if there are equal numbers of negative and positive slopes across the 25 phyla.

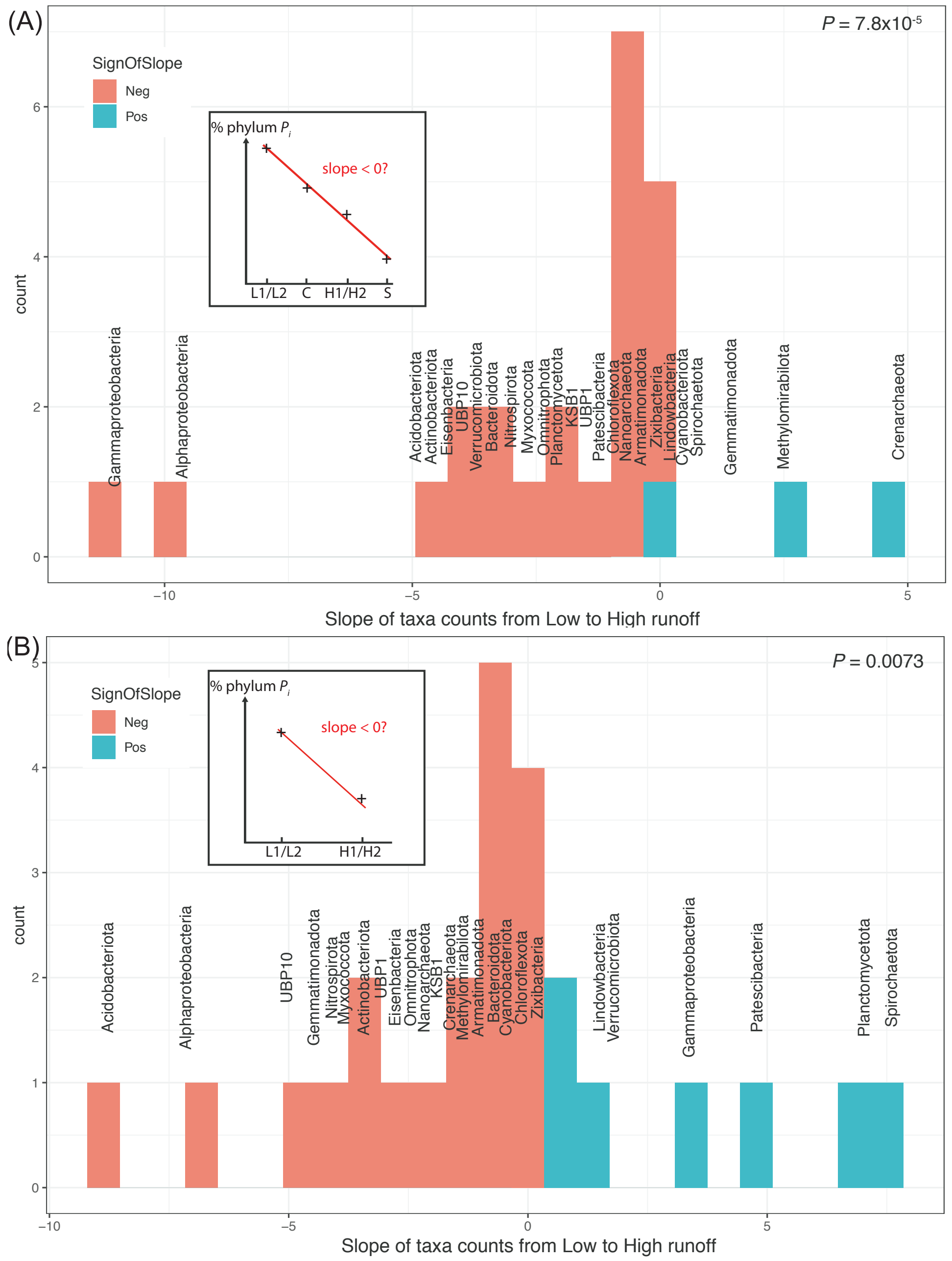

Supplementary Figure 8. Transition from low to high runoff leads to a decrease in diversity. (A) Distribution of the slopes of taxonomic counts as a function of sites across all four sites ((L [Low], C [Control], S [Soil], and H [High] sites); same as Figure 4A). (B) Distribution of the slopes of taxonomic counts as a function of sites across L and H sites only. In both cases, linear models (ANOVA) were fitted to estimate the slope of each regression. P-values: one-sided binomial test for enrichment in negative slopes across the 25 phyla, whose names are shown in black. Note that the scales on the x-axes differ. Insets show the design, as in Supplementary Figure 6.

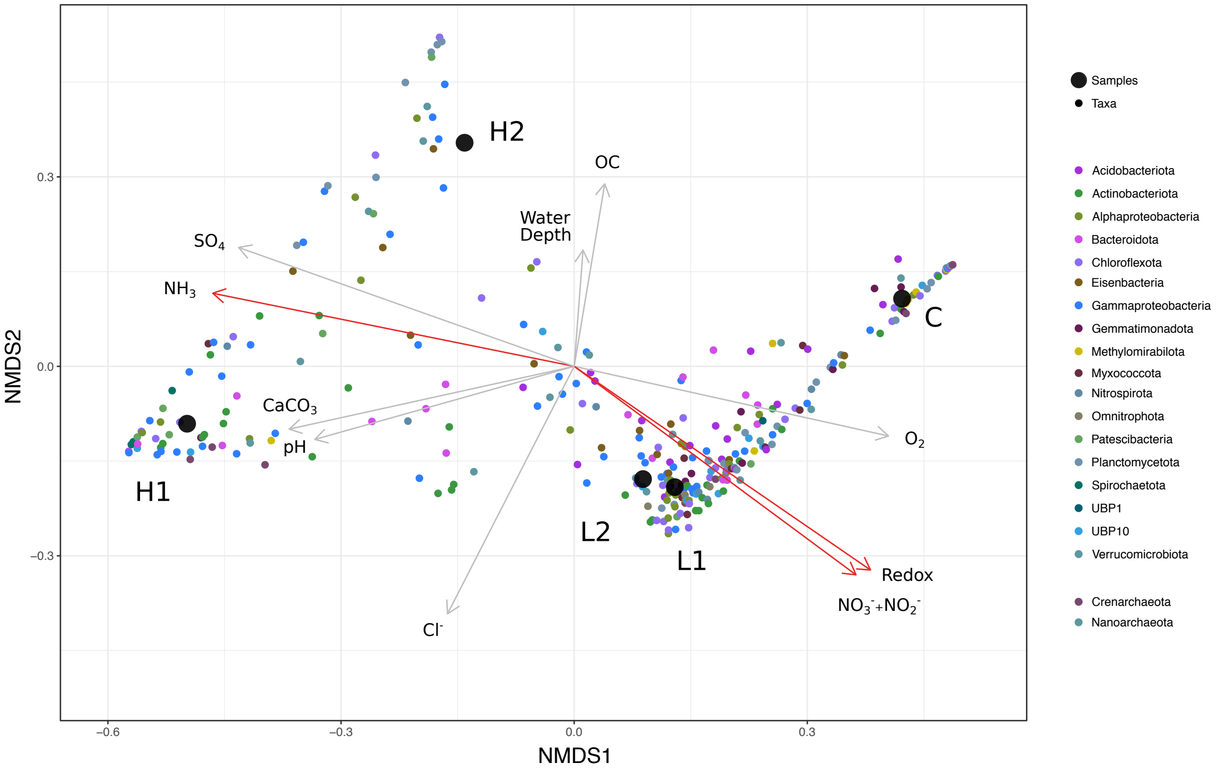

Supplementary Figure 9. Non-metric Multi-dimensional Scaling (NMDS) of genomes from the 20 most abundant taxa. Taxa are shown as small colored dots and grouped at a phylum level except for Proteobacteria (which are grouped at the class level). Sediment sites are shown as larger black dots. Physical and chemical vectors are fitted to data. Red vectors are significant chemical parameters (permutation test: $P < 0.05$). The corresponding R^2 values and P-values are redox 0.9915, 0.03333; NH_3 0.9743, 0.02500; and $NO_2^- + NO_3^-$ 0.9847, 0.02500.

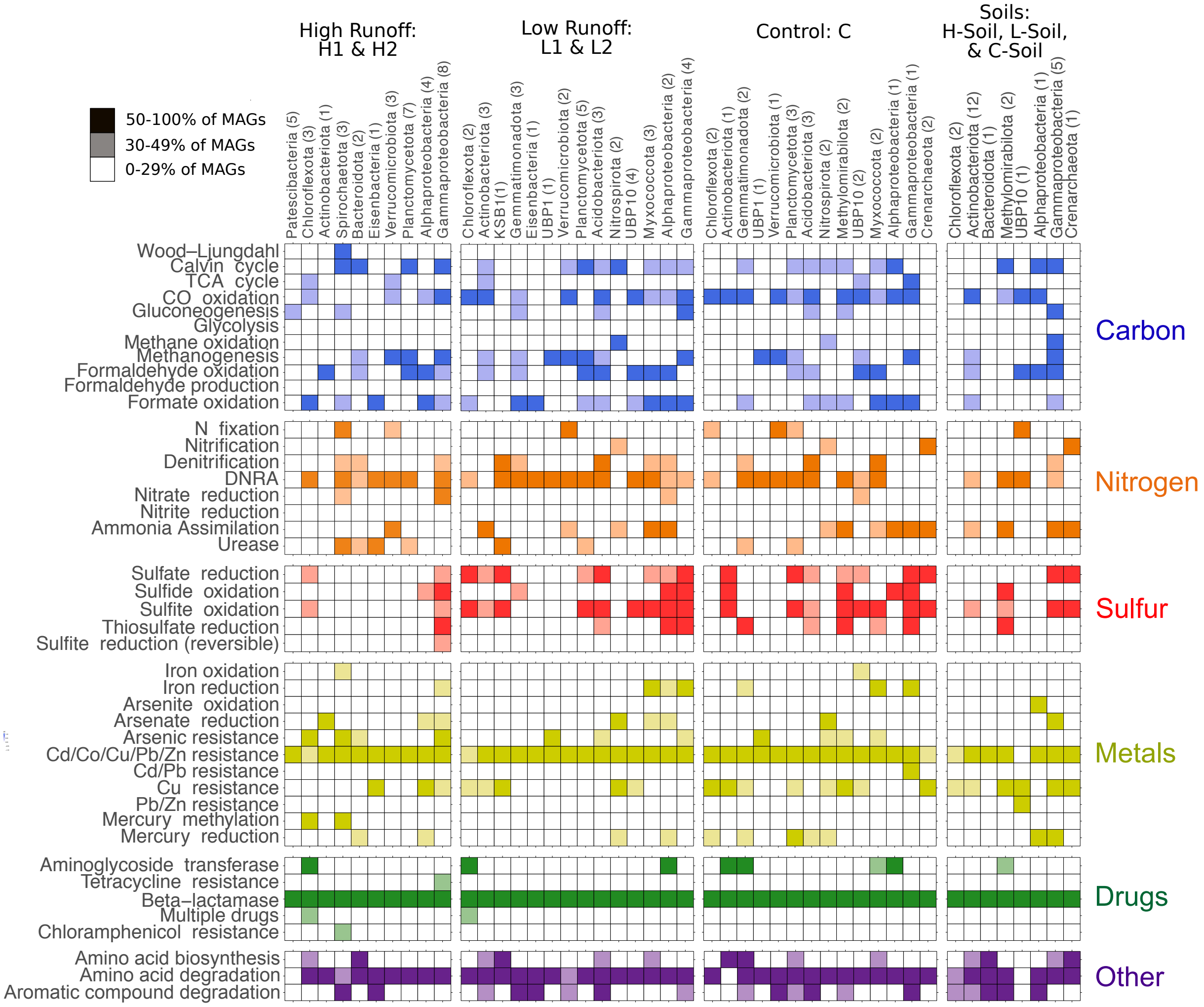

Supplementary Figure 10. Metabolic capacity of genomes separated by hydrological regime. Genomes are only considered to contribute to a site if 0.56% ($-\log_{10} \leq 0.25$) of reads per sample mapped to a genome. Presence of core metabolic genes involved in carbon metabolism, nitrogen metabolism, sulfur metabolism, metal cycling, antibiotic resistance, and other metabolism are shown. Number of genomes for each taxa are shown in parentheses. Blank: genes predicting function are absent or in low abundance. Shaded colors: genes predicting function are present in 30-50% of genomes per phylogenetic group. Dark colors: genes predicting function are present in >50% of genomes per phylogenetic group.

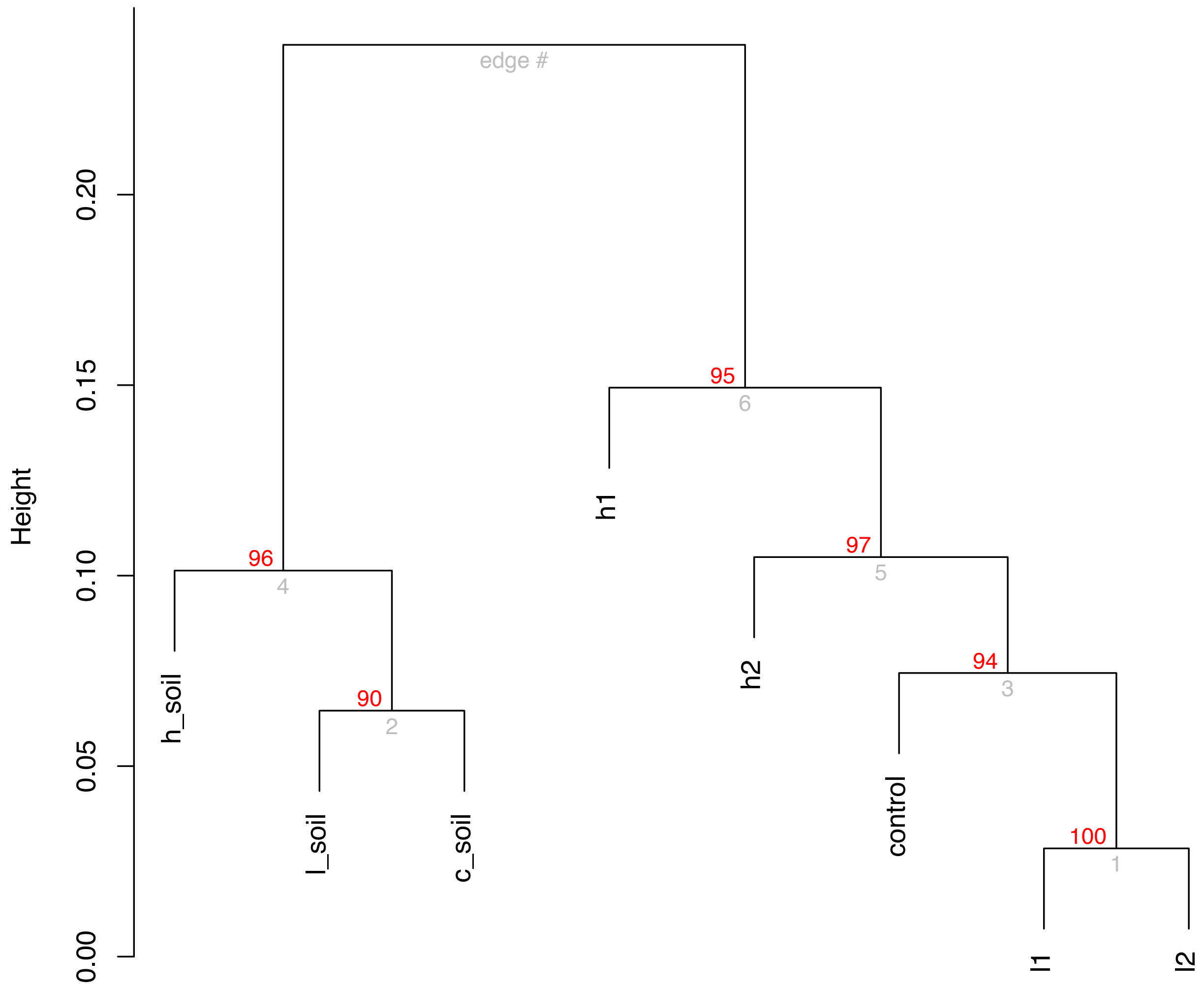

Supplementary Figure 11. Metabolic capacity for carbon, sulfur, and nitrogen cycles clustered with P-values via multiscale bootstrap resampling. AU (approximately unbiased) P-values are shown in red, with any value > 0.95 (significance level 0.05) being significant. Hierarchical clustering was completed using "correlation" as distance method and "average" as clustering method.

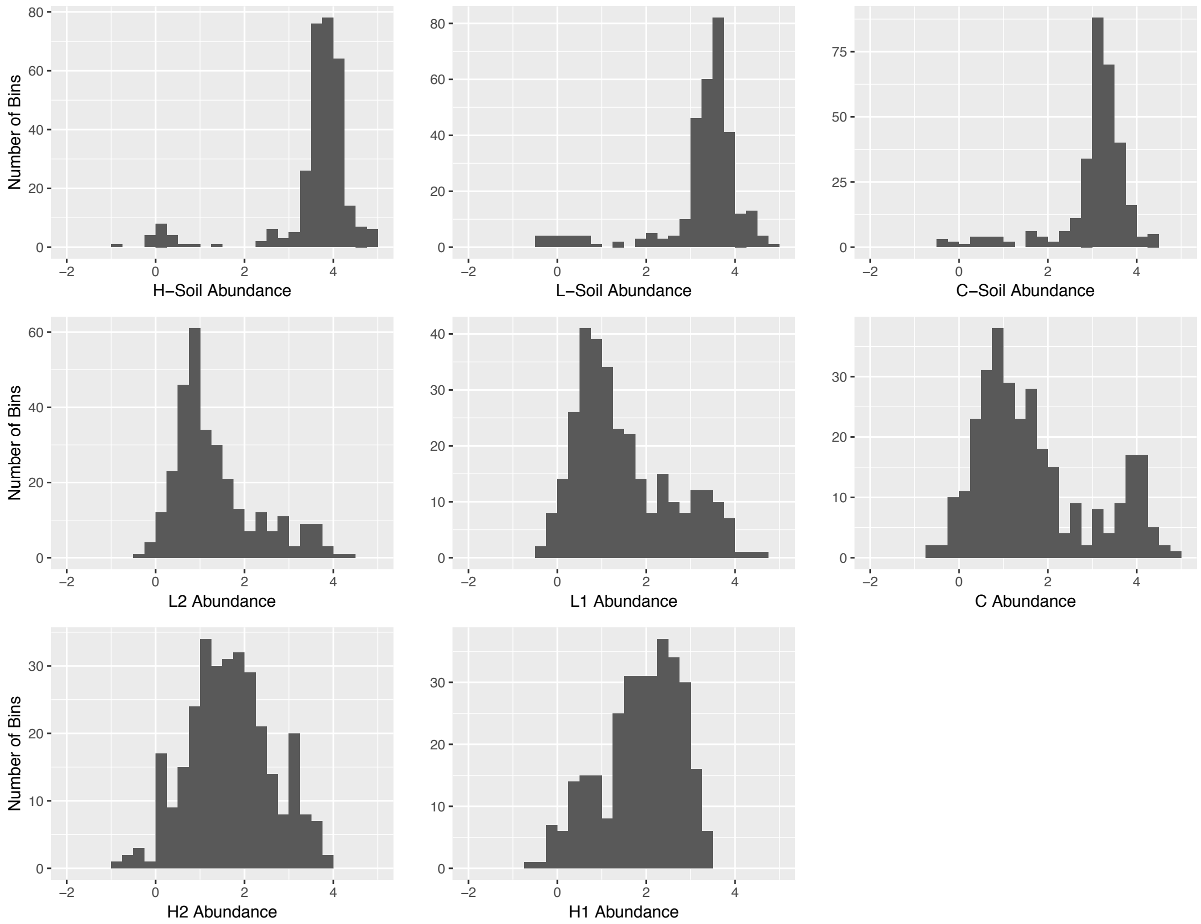

Supplementary Figure 12. Genome abundance per sample ($-\log_{10}$ scale).

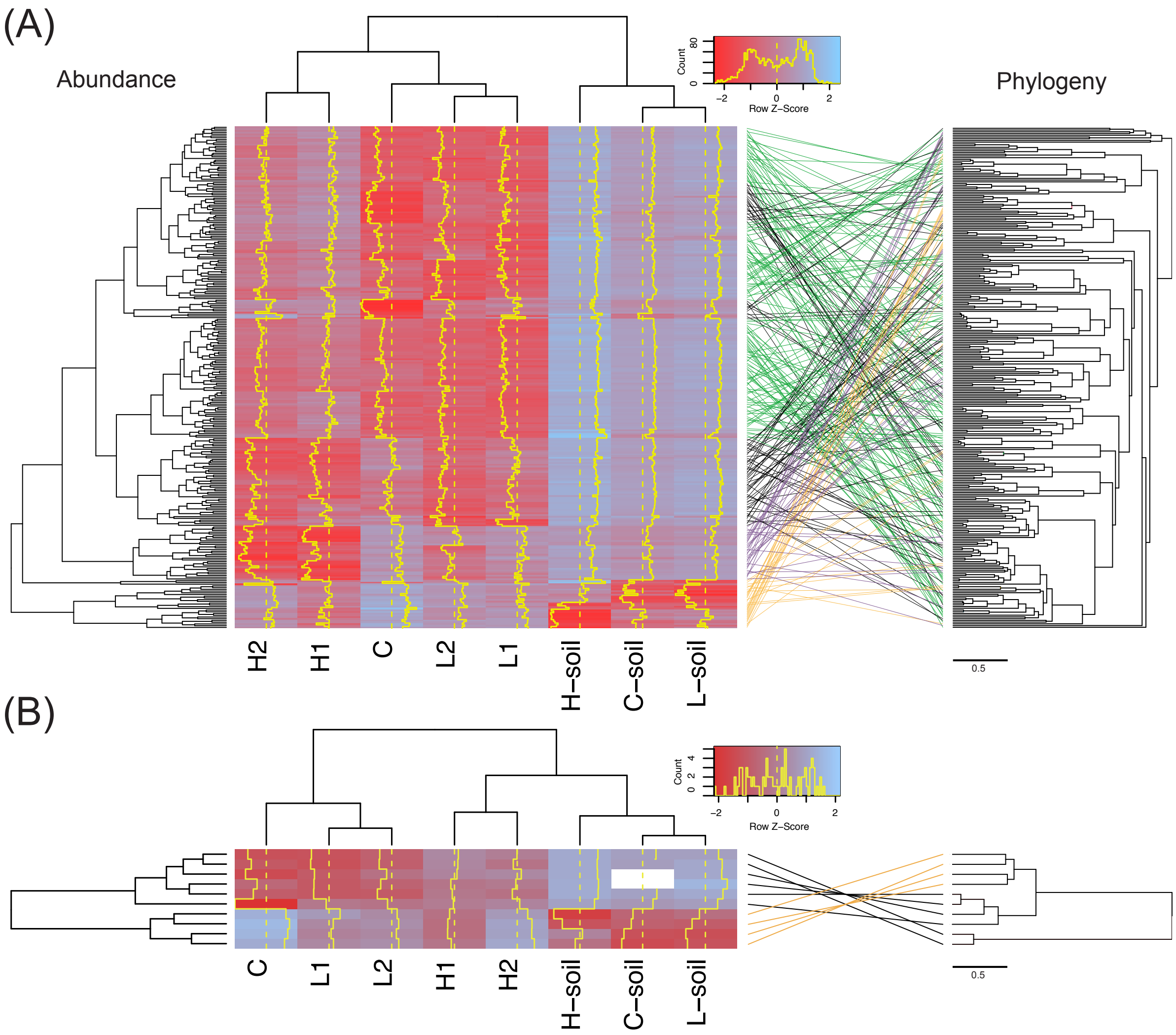

Supplementary Figure 13. Genome abundance heatmap and tanglegram separated by (A) bacterial genomes and (B) archaeal genomes. Left: the heatmap displays genome abundance per site normalized by amount of reads in each sample and transformed to a $-\log_{10}$ scale. Dotted yellow lines represent mean abundance values, and yellow traces represent the raw z-scores above (red) and below (blue) the mean. The abundance values are grouped both by sites (top dendrogram) and genomes (left dendrogram). Right: tanglegram between dendrogram clustered by similar abundances and phylogenetic tree. Highlights in tanglegram: orange lines are genomes abundant in soil, green lines are genomes abundant in low runoff sediment, purple lines are genomes abundant in high runoff sediment, black lines are genomes shared in multiple environments.

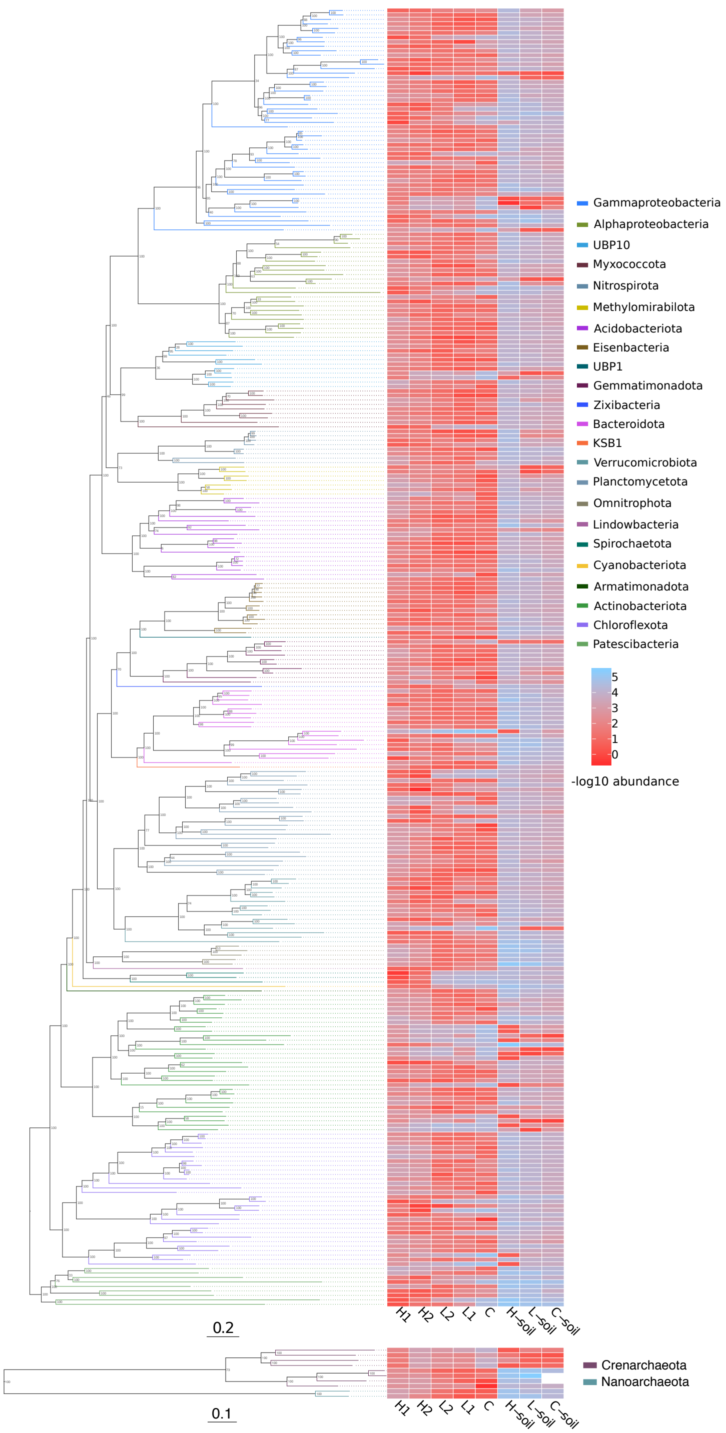

Supplementary Figure 14. Phylogenetic distribution and abundance of all 300 MAGs. MAGs are presented on a $-\log_{10}$ scale, where more negative values (red) are more abundant than positive values (blue). The abundance values correspond by row with the genomes phylogenetic assignment. Support values for phylogenetic tree are shown at each node.

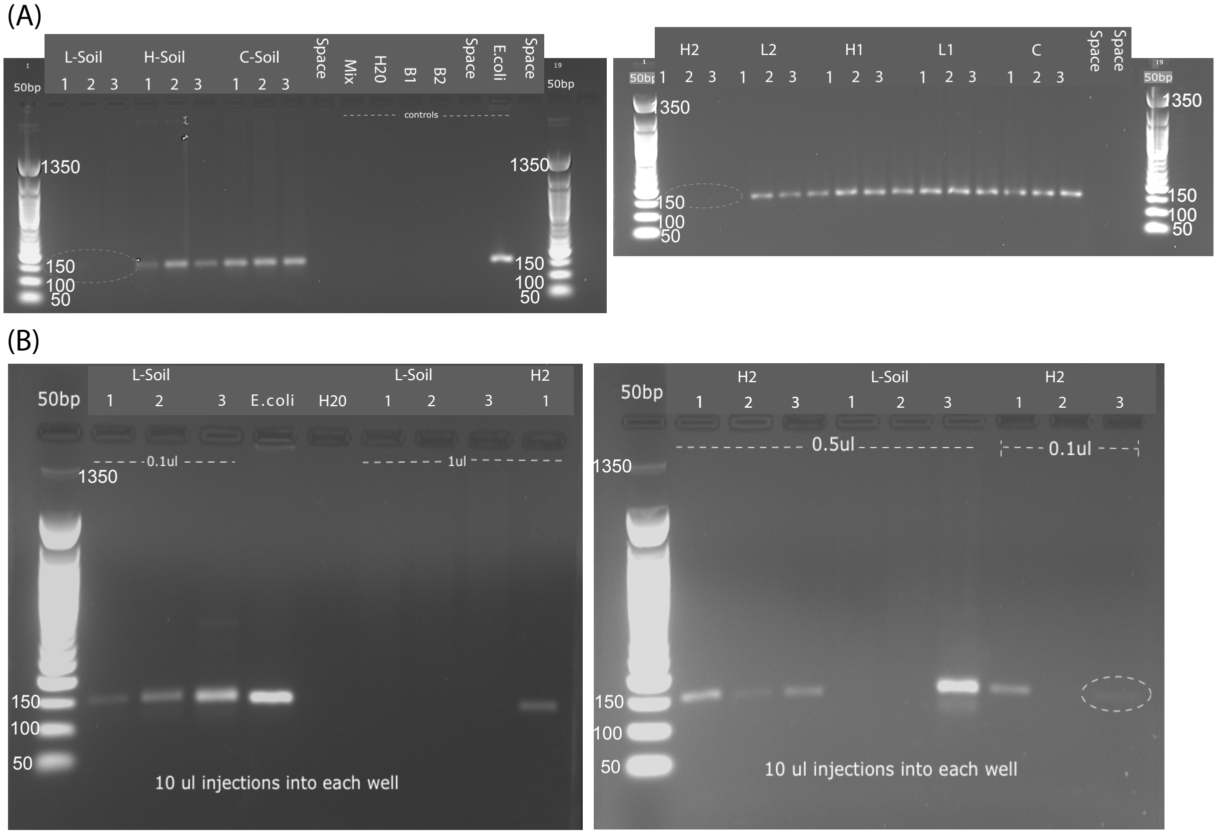

Supplementary Figure 15. Gel electrophoresis images of the *glnA* gene for each sample extracted in triplicate. (A) Initial gel results using 1 μl of DNA for each PCR reaction. Every sample contains *glnA* except L-Soil and H2. (B) Repeated PCR reaction for L-Soil and H2. Diluted DNA concentrations for PCR from 1 μl to 0.1 μl and 0.5 μl.

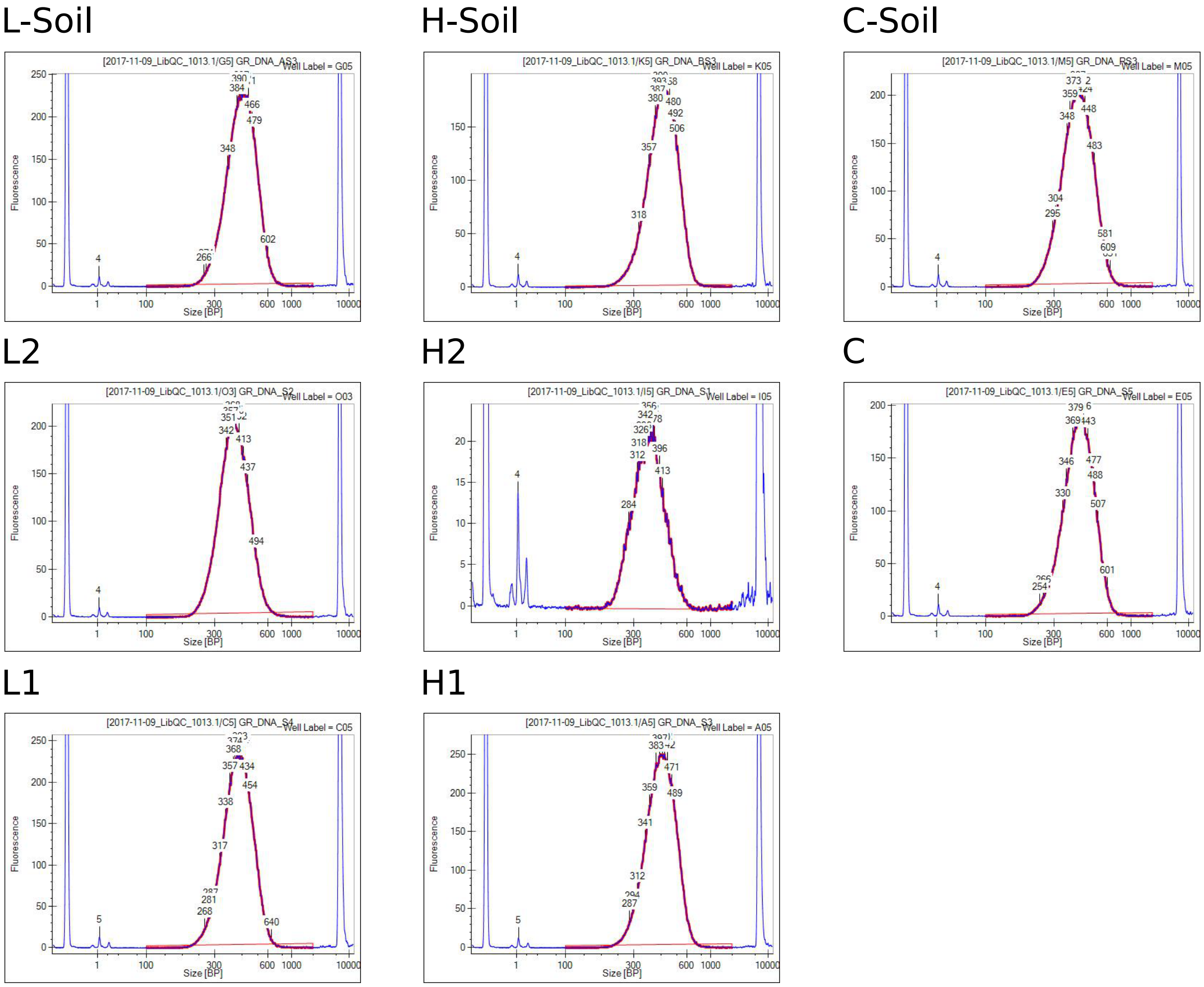

Supplementary Figure 16. Illumina HiSeq DNA sequencing library validation from an Agilent Technology 2100 Bioanalyzer. All libraries were constructed using Illumina TruSeq DNA PCR-Free Library Prep. The library results for each of the eight samples is presented and labeled above the panel: L-Soil, H-Soil, C-Soil, L2, H2, C, L1, H1. Peaks between 300-600 bp indicate the presence of DNA. Peaks below 1 bp and above 1000 bp are internal standards.

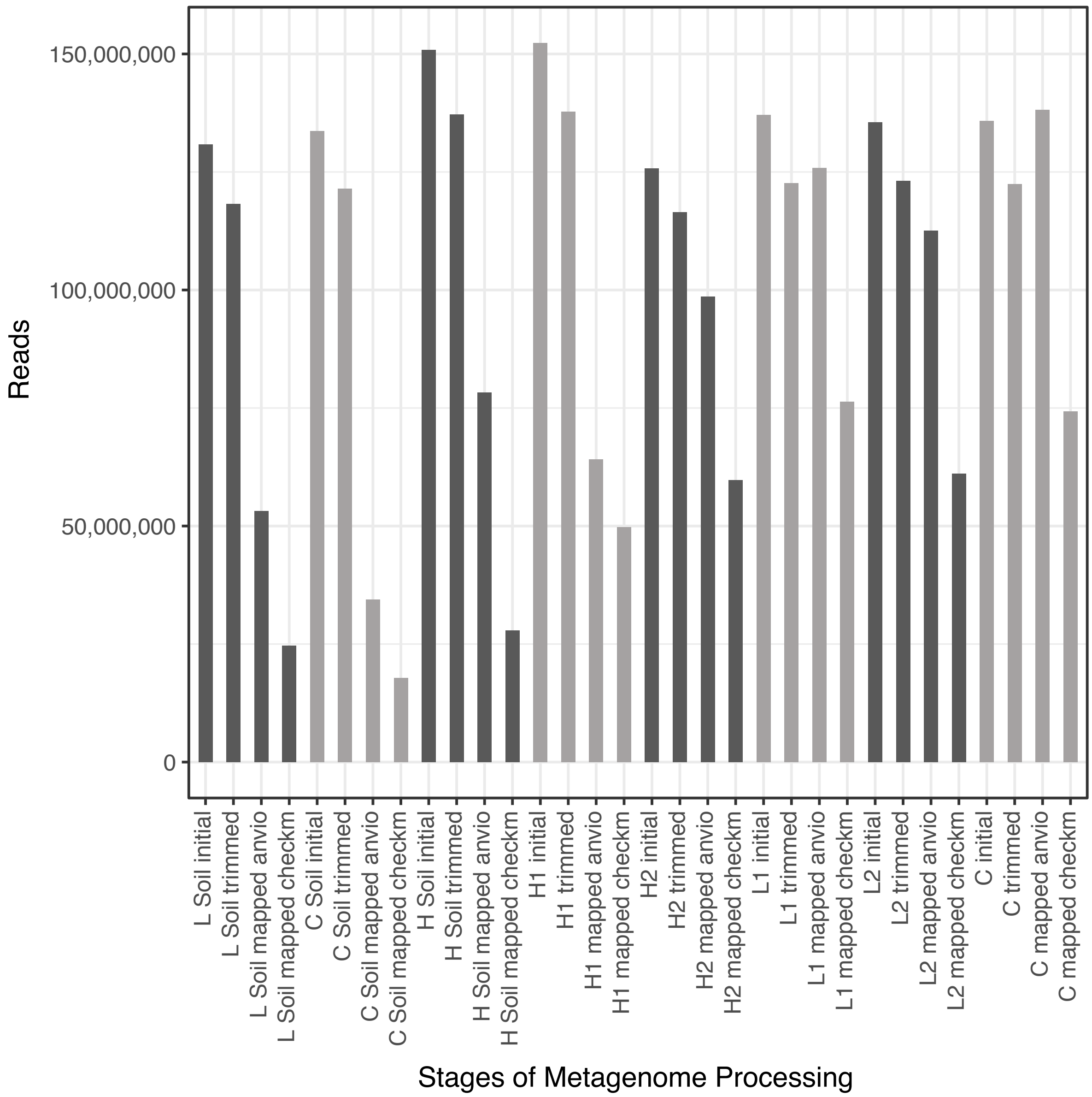

Supplementary Figure 17. Number of reads throughout analysis. Reads are successively decreasing, except for the reads mapped to the Anvio contig database, as each read can map to more than one contig and be counted more than once. The reads mapped using checkM include only reads that map to the 300 high quality reconstructed genomes, opposed to all reconstructed genomes in the Anvio mapping.

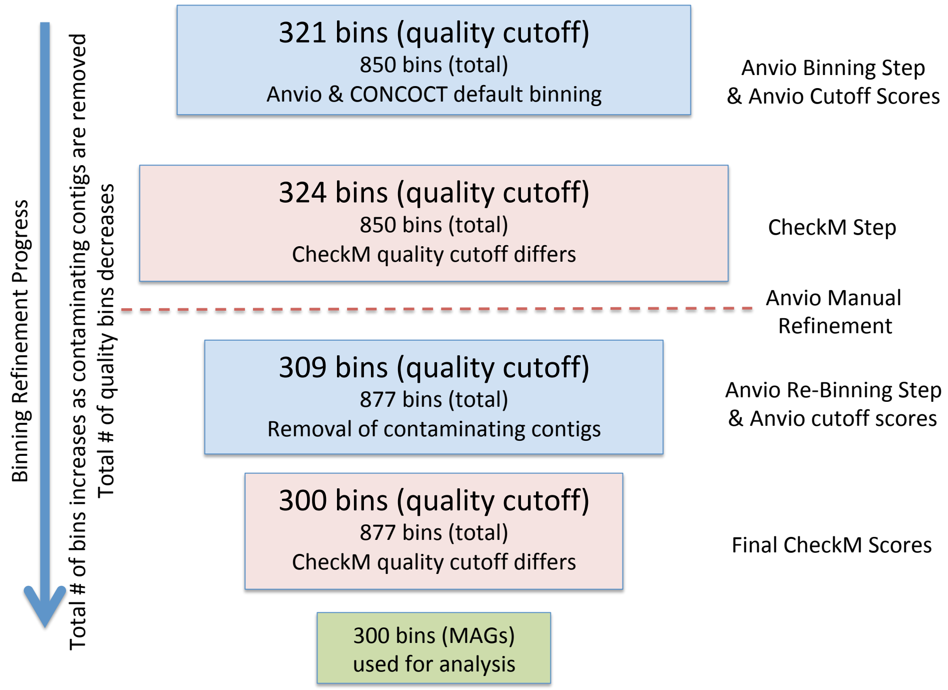

Supplementary Figure 18. Flow chart of the binning procedure. The binning of contigs relied on an iterative process of binning, removing contaminated contigs from bins, and re-binning. The "Manual Refinement" step, or removal of contaminated contigs from bins, was completed on the basis of GC content, differential abundance in samples, and taxonomy of contigs assigned by Kaiju.

Supplementary tables

Supplementary Table 1. Contigs were assembled with Megahit and used in Anvio database. Only contigs that were longer than 2500 bp were used to form MAGs, even if contigs with a length $\geq$ 1000 bp were used to form the initial database.

	Contigs	Total Length (bp)	Min Contig Length (bp)	Max Contig Length (bp)	Avg Contig Length (bp)	N50 (bp)	# of genes prodigal
**Megahit**	12026467	8477069127	200	792468	705	756	NA
**Anvio**	1455655	3414974759	1000	792468	NA	285024	4254625

Supplementary Table 2. Number of bins recovered at each assembly step prior to and after manual refinement with Anvio.

	**Anvio-Output (CONCOCT binning)**	**CheckM**	**Anvio-Output (manually refined)**	**Final CheckM**
Total Bins	850	850	877	877
Completion, Redundancy				
C: >90, R: <10	52	74	52	72
C: >70, R: <10	180	199	178	198
C: >50, R: <10	321	324	309	300
C: <50, R: any	529	526	568	577
Contamination				
<1	233	261	255	286
<5	544	632	590	676
>10	8	51	7	27
Good bins but contaminated				
C: >50, R: >10	2	27	0	2
C: >70, R: >10	1	6	0	0
C: >90, R: >10	0	2	0	0

Supplementary Table 3. P-values for marker gene and pathway distribution between sites. A Fisher's exact test was used in the place of χ^2 test for small counts. P-values ≤ 0.05 are considered significant (indicated with an asterisk).

Functional Marker	High vs Low	High vs Control	High vs Soil	Low vs Control	Low vs Soil	Control vs Soil
Carbon Cycle	0.03298 *	0.0004998 *	0.0004998 *	0.07746	0.1384	0.001499 *
Nitrogen Cycle	0.07596	0.001999 *	0.0004998 *	0.7006	0.03448 *	0.1859
sulfur Cycle	0.001999 *	0.002999 *	0.0004998 *	0.4913	0.02999 *	0.3823

Supplementary Table 4. Coordinates of Lake Hazen sediment and soil sites with temperature and date at time of sampling.

Sample	Location	Temperature (°C)	Date
**H-Soil**	81° 84’ 840” N; 70° 83’ 849” W	-5	June 3, 2017
**L-Soil**	81° 80’ 332” N; 71° 54’ 239” W	0	June 7, 2017
**C-Soil**	81° 79’ 382” N; 70° 44’ 486” W	-2	June 3, 2017
**H1**	81° 84’ 150” N; 70° 85’ 175” W	not measured	May 24, 2017
**H2**	81° 82’ 493” N; 70° 71’ 498” W	not measured	May 29, 2017
**C**	81° 80’ 343” N; 70° 50’ 447” W	not measured	May 27, 2017
**L1**	80° 80’ 521” N; 70° 52’ 699” W	not measured	June 1, 2017
**L2**	81° 79’ 171” N; 71° 46’ 926” W	not measured	June 2, 2017

Supplementary Table 5. Glacial runoff to the Lake Hazen watershed. Lengths of rivers in km are shown in parentheses. Mass balance modeled runoff for years 2015 and 2016. Sampling dates in 2017 were prior to the summer runoff. Data adapted from previous study [10].

	**Surface area (km^2)**			**Runoff volume (km^3)**	
**River**	**Catchment**	**Glacier**	**River**	**2015**	**2016**
High Runoff: Abbé (AB)	390	204	7.9 (21)	0.061	0.015
Low Runoff: Blister (BR)	n/a	6	2.5 (11)	0.002	<0.001
Gilman (GL)	992	708	5.1 (22)	0.192	0.08
Henrietta Nesmith (HN)	1274	1041	9.6 (4.6)	0.291	0.075
Snowgoose (SG)	222	87	5.6 (17)	0.026	0.006
Turnabout (TN)	678	259	13.4 (42)	0.082	0.024
Very (VR)	1035	269	32.9 (39)	0.165	0.08
Watershed total	7516	3078	91.2	0.979	0.291

Supplementary Table 6. Sediment deposition dates and rates for the two deep sites, H2 and L2, is based on ^{210}Pb constant rate of supply (CRS) dating model. Analysis was completed on 0.5 cm core intervals. Data adapted from previous study [10].

H2 (Abbe Deepsite)			L2 (Blister Deep Site)		
**Interval**	**Midpoint CRS date (CRS year)**	**Sedimentation rate (g/cm^2/yr)**	**Interval**	**Midpoint CRS date (CRS year)**	**Sedimentation rate (g/cm^2/yr)**
0-0.5	2017.1	0.349	0-0.5	2016.5	0.0724
0.5-1	2016.6	0.292	0.5-1	2014.0	0.0701
1-1.5	2015.9	0.111	1-1.5	2011.3	0.1163
1.5-2	2014.1	0.671	1.5-2	2009.2	0.1759
2-2.5	2012.7	0.671	2-2.5	2007.3	0.1232
2.5-3	2012.7	0.227	2.5-3	2004.7	0.0909
3-3.5	2012.0	5.563	3-3.5	2001.9	0.0967
3.5-4	2011.3	5.563	3.5-4	1998.8	0.0854
4-4.5	2011.3	5.563	4-4.5	1994.0	0.0513
4.5-5	2011.3	5.563	4.5-5	1987.2	0.0375
5-5.5	2011.3	5.563	5-5.5	1976.7	0.0220
5.5-6	2011.3	5.563	5.5-6	1966.51	0.0515
6-6.5	2011.3	5.563	6-6.5	1957.66	0.0339
6.5-7	2011.3	5.563	6.5-7	1949.00	0.0537
7-7.5	2011.3	5.563	7-7.5	1940.41	0.0451
7.5-8	2011.3	5.563	7.5-8	1931.79	0.0668
8-8.5	2011.3	5.563	8-8.5	1925.07	0.0553
8.5-9	2011.3	0.441	8.5-9	1918.10	0.0553
9-9.5	2010.9	0.224	9-9.5	1911.08	0.0553
9.5-10	2009.8	0.123	9.5-10	1904.42	0.0553

Supplementary Table 7. DNA extraction masses. DNA was extracted in triplicate for each sample and then combined prior to sequencing.

Lake Hazen	Sample Location	Tube ID	Wet Weight (g)				PCR with *glnA*
			1	2	3	Total (grams)	
Sediment	H2: Deephole	S1	0.416	0.455	0.499	1.370	yes (diluted)
	L2: Blister Deep	S2	0.497	0.455	0.389	1.341	yes
	H1: Abbe	S3	0.469	0.514	0.552	1.535	yes
	L1: Blister Shallow	S4	0.429	0.402	0.361	1.192	yes
	C: Ruggles	S5	0.518	0.447	0.331	1.296	yes
Soil	L-Soil: Blister Soil	BS3	0.527	0.535	0.561	1.623	yes (diluted)
	H-Soil: Abbe Soil	AS3	0.443	0.537	0.417	1.397	yes
	C-Soil: Ruggles Soil	RS3	0.447	0.500	0.447	1.394	yes

Supplementary Table 8. DNA fluorescence assay quantification for each sample. Note: H2 sequencing required using full extraction volume of 65 μl to reach an appropriate concentration.

Sample	Volume (μl)	Concentration (ng/μl)	Total DNA (ng)	NanoDrop (concentration ng/μl)
H2	65	0.08	3.36	4.6
L2	42	2.55	107.1	7.6
H1	42	4.33	181.86	10.2
L2	42	17.98	755.16	32.8
C	42	21.2	890.4	36.4
H-Soil	42	38.14	1601.88	65.2
L-Soil	47	69.88	3284.36	149.0
C-Soil	47	74.38	3495.86	110.0
